# Optimizing nitrogen fertilization in maize: the impact of nitrification inhibitors, phosphorus application, and microbial interactions on enhancing nutrient efficiency and crop performance

**DOI:** 10.3389/fpls.2024.1451573

**Published:** 2024-10-02

**Authors:** Ali Malakshahi Kurdestani, Davide Francioli, Reiner Ruser, Alessandro Piccolo, Niels Julian Maywald, Xinping Chen, Torsten Müller

**Affiliations:** ^1^ Institute of Crop Science, University of Hohenheim, Stuttgart, Germany; ^2^ Department of Soil Science and Plant Nutrition, Hochschule Geisenheim University, Geisenheim, Germany; ^3^ Department of Agricultural Sciences, University of Naples Federico II, Via Università, Portici, Italy; ^4^ College of Resources and Environment, Academy of Agricultural Sciences, Southwest University, Chongqing, China

**Keywords:** nitrification inhibitors (NIs), phosphorus acquisition, DMPFA (Dimethyl pyrazole fulvic acid), nutrient uptake, soil microbial activity

## Abstract

Despite the essential role of nitrogen fertilizers in achieving high crop yields, current application practices often exhibit low efficiency. Optimizing nitrogen (N) fertilization in agriculture is, therefore, critical for enhancing crop productivity while ensuring sustainable food production. This study investigates the effects of nitrification inhibitors (Nis) such as Dimethyl Pyrazole Phosphate (DMPP) and Dimethyl Pyrazole Fulvic Acid (DMPFA), plant growth-promoting bacteria inoculation, and phosphorus (P) application on the soil-plant-microbe system in maize. DMPFA is an organic nitrification inhibitor that combines DMP and fulvic acid for the benefits of both compounds as a chelator. A comprehensive rhizobox experiment was conducted, employing varying levels of P, inoculant types, and Nis, to analyze the influence of these factors on various soil properties, maize fitness, and phenotypic traits, including root architecture and exudate profile. Additionally, the experiment examined the effects of treatments on the bacterial and fungal communities within the rhizosphere and maize roots. Our results showed that the use of Nis improved plant nutrition and biomass. For example, the use of DMPFA as a nitrification inhibitor significantly improved phosphorus use efficiency by up to 29%, increased P content to 37%, and raised P concentration in the shoot by 26%, compared to traditional ammonium treatments. The microbial communities inhabiting maize rhizosphere and roots were also highly influenced by the different treatments. Among them, the N treatment was the major driver in shaping bacterial and fungal communities in both plant compartments. Notably, Nis reduced significantly the abundance of bacterial groups involved in the nitrification process. Moreover, we observed that each experimental treatment employed in this investigation could select, promote, or reduce specific groups of beneficial or detrimental soil microorganisms. Overall, our results highlight the intricate interplay between soil amendments, microbial communities, and plant nutrient dynamics, suggesting that Nis, particularly DMPFA, could be pivotal in bolstering agricultural sustainability by optimizing nutrient utilization.

## Introduction

In the intricate realm of soil fertility and nutrient management, comprehending the dynamics of phosphorus (P) acquisition is essential for optimizing agricultural productivity. Plant growth and development rely on phosphorus, which is vital for biochemical processes like energy transfer and nucleic acid synthesis. The inherent immobility and tendency to form insoluble compounds limit the availability of phosphorus in the soil. Thus, phosphorus fertilization is a common practice in modern agriculture. In most soils, phosphates that are easily accessible to plants typically make up less than 0.1% of the total soil phosphorus (Menezes-Blackburn et al., 2018). From 1961 to 2013, the nutrient utilization efficiency (NUE) for phosphorus fertilizers was estimated to be 16% globally (Dhillon et al., 2017). This low P efficiency is due to the strong tendency of soils to fix phosphorus, necessitating high application rates to maintain consistent yields, which in turn increases the risk of over-fertilization and raises production costs.

In the case of N fertilization, ammonium-based fertilizers have a stronger affinity for soil particles, thereby reducing the risk of N leaching, a common issue with nitrate fertilizers. However, in aerated soils, ammonium is quickly converted by nitrification into nitrate, which is highly mobile in most soils and can be easily leached. To address this issue, a method of choice involves chemically stabilizing ammonium fertilizers or ammonium-rich organic fertilizers using nitrification inhibitors (Nis) (Nkebiwe et al., 2016a). Furthermore, ammonium fertilization can additionally improve the acquisition of sparingly soluble nutrients, such as P, Fe, Zn, and Mn, due to rhizosphere acidification, a process induced by preferential ammonium uptake by the plant and enhanced nitrification (Marschner and Romheld, 1983; Neumann and Römheld, 2003). This effect can be further improved by localized application of P combined with ammonium fertilizers, leading to the formation of rhizosphere hot spots by local proliferation of lateral roots as an effective management strategy for improving nutrient use efficiency (Jing et al., 2010). In soils with lower pH, the same effect may be exploited to improve the acquisition of acid-soluble P fertilizers, such as rock-phosphate (RP) or P fertilizers based on products of waste recycling.

A sustainable approach to enhance plant nutrition and fitness involves the utilization of beneficial microbial inoculants. This methodology is gaining prominence in response to escalating pressure to adopt environmentally friendly agricultural practices. The transition toward more sustainable farming practices holds the potential to augment biodiversity, enhance soil resilience, and promote overall soil health. Plant growth promoting microorganisms (PGPM) have demonstrated the ability to stimulate plant growth through diverse mechanisms, including the modulation of phytohormone levels (Zubair et al., 2019), increased nutrient availability (Behr et al., 2023), and the induction of systemic resistance to protect plants against both below- and aboveground pathogens (Vlot et al., 2021). Concerning various nitrogen fertilization approaches, the stimulatory effects of stabilized ammonium fertilization on beneficial microorganisms employed as plant inoculants (such as selected strains of *Pseudomonas*, *Bacillus*, *Paenibacillus*, and *Trichoderma*) have been reported in both pot experiments and under field conditions, specifically in maize and tomato cultivation. These effects manifest in terms of enhanced root colonization and the promotion of root growth (Nkebiwe et al., 2016b; Mpanga et al., 2019a, b), leading to a more robust acquisition of nutrients, particularly P (Bradáčová et al., 2019; Mpanga et al., 2019a, b).

Recent research has further recognized the effect of humic acids on improving physio-chemical properties, microbial diversity, and enzyme activities in soils (Sharma et al., 2013; Pukalchik et al., 2019). Similar to beneficial microbes, humic acids and fulvic acids released from lignin-containing organic matter (Aggag et al., 2015), can act as efficient biostimulants, improving plant stress resistance and nutrient acquisition by stimulation of plasmalemma ATPases involved in nutrient uptake and root growth stimulation, solubilization of micro and macro elements, decreased active levels of toxic minerals and increased microbial populations (Yang et al., 2013; Canellas et al., 2015).

In 2021, DMPFA was designed by Malakshahi Kurdestani at the University of Hohenheim (Germany). This compound, which consists of DMP and fulvic acid (FA), functions as a double chelator. Due to their chelating properties, fulvic acids may also contribute to the inhibition of nitrification by the formation of metal complexes, with Cu and Fe otherwise acting as enzymatic co-factors for microbial nitrifiers. The DMPFA patent registration is currently being processed. The type of bonding between FA and DMP are previously reported (Mazzei et al., 2022).

This study investigates the effects of Nis and their interactions with bacteria on P acquisition in conditions where P is sparingly soluble. To evaluate the impact of various agricultural practices, including Nis application, P fertilization, and microbial inoculation, we conducted a greenhouse experiment. In this experiment, maize plants were cultivated in low P soil enriched with RP, Nis, and inoculated with *Bacillus atrophaeus*, with a control group included for comparison.

We hypothesized that Nis can enhance plant nutrient uptake and increase P availability in low P soils by triggering rhizosphere processes and promoting soil acidification, with a differential effect depending on the specific Nis employed. Additionally, we hypothesized that these processes would significantly alter the microbial community structure in the maize rhizosphere, as changes in soil properties induced by the various experimental treatments would be strongly correlated with shifts in microbial assemblage.

## Materials and methods

Starting from July 10th, 2022, our rhizobox experiment lasted for six weeks. A low P silt loam soil was used for this experiment. According to the VDLUFA method, the extractable P content with P-CAL was 8.7 mg kg^-1^, indicating class A (very low, VDLUFA, 2018). The pH of 6.4 in 10^-2^ M CaCl_2_ solution reflected near-neutral soil conditions, promoting optimal plant growth. The organic carbon concentration (C_org_) was 2.2% and total N concentration (N_t_) 0.02%. The substrate was a mixture of 70% soil and 30% quartz (w/w). The substrate was fertilized with P as RP: 150 mg kg^-1^ dry matter (DM); K as K_2_SO_4_: 150 mg kg^-1^DM. Nitrogen as ammonium and ammonium plus nitrification inhibitors was applied at a rate of 150 mg kg^-1^ DM.

The experiment was designed as 3-factorial experiment: (1) N fertilization in three levels, (2) P fertilization in 2 levels, and (3) inoculation in 2 levels. The experimental design followed a completely randomized design, with four replicates per treatment. In the experiment, three different N sources were used: (1) Ammonium sulfate (NH_4_)_2_SO_4_ as control, (2) Novatec^®^ solub 21 ((NH_4_)_2_SO_4_ stabilized with DMPP (3,4-dimethylpyrazole phosphate)), and (3) Ammonium sulfate plus DMPFA ((NH_4_)_2_SO_4_ stabilized with 3,4-dimethylpyrazole fulvic acid). P fertilization was used in two levels: (1) rock phosphate (Dolophos 26^®^ rock phosphate (11% P, 1.8% Mg)), and (2) control (without P application). Two levels of inoculation were employed: (1) *Bacillus atrophaeus* (RhizoVital^®^ C5), and (2) control (without inoculation).

Each rhizobox contained 2 kg of substrate, watered to 65% of its maximum water-holding capacity. Three maize seeds (*Zea mays* var. *Ronaldinio*) were sown beneath the substrate surface, and after emergence, the plants were carefully thinned to one per pot.

The rhizoboxes were placed in a greenhouse at the University of Hohenheim, Stuttgart, Germany. The temperature ranged from 13 to 32°C throughout the growing period. Maize plants were harvested 42 days after planting.

### DMPFA

DMPFA designed based on 40% m/v 3,4-Dimethyl-1H-pyrazol (CAS-Nummer: 2820-37-3), 20% v/v 2-Propanol as a solvent and 10 g/L fulvic acid. First, 2-Propanol was added to 3,4-Dimethyl-1H-pyrazole and the new solution was left for ten minutes at room temperature. Then fulvic acid was added, and the solution was placed on a magnetic plate and stirred for three hours at 40 Celsius degrees (**Supplementary Figure S1**). The structure of the used fulvic acid is described in García Díaz et al. (2015).

### Inoculation

RhizoVital^®^ C5 (*Bacillus atrophaeus* ABi05; (Pro: 1x10^8^ CFU kg^-1^ substrate)) was utilized as a PGPM. Prior to sowing, maize seeds were soaked in the RhizoVital^®^ C5 suspensions for one hour. The microbial interaction continued after the seeds were planted. We carefully inoculated 20 mL of the *B. atrophaeus* suspension into the seeding hole, promoting a localized and ongoing interaction between the plant roots and the PGPM used in this study. The aim of this approach was to create a mutually beneficial connection between the plant and *B.atrophaeus* ABi05, potentially improving nutrient absorption and overall plant well-being. In order to sustain this plant-microbial partnership, we introduced a thoughtful routine of two additional weekly applications of the PGPM suspension close to the plant stem.

### Analysis of nutrient analysis and use efficiency calculation

The plant mineral nutrient status of the dried plant material (root, aboveground) was assessed by using an established wet-chemical extraction method after grinding the plant dry matter (VDLUFA, 2011). P concentration in the extract was measured at 436 nm using vanadate/molybdate as a color agent in the spectrophotometer (Gericke and Kurmis, 1952). K was measured by flame emission photometry and Mn, Zn, Fe, and Mg were determined by atomic absorption spectrometry. Phosphorus and nitrogen use efficiency (PUE and NUE) was calculated by using Equations 1 and 2, respectively.

Equation 1:


(1) $$PUE\left(\%\right)=\frac{P\;content\;mg\;per\;plant}{Input\;P\;mg\;as\;fertilizer\;per\;plant}*100$$

Equation 2:


(2) $$NUE\left(\%\right)=\frac{N\;content\;mg\;per\;plant}{Total\;mg\;Nmin\;in\;the\;soil}*100$$

### Assessment of biomass, root length, and rhizosphere pH

During the final harvest after six weeks, maize plants were carefully removed from the rhizoboxes, and bulk soil was removed by shaking the plants. Soil still adhering to the roots was then sampled with a brush and defined as rhizosphere soil. The VDLUFA (1991) method was used to determine the soil pH. The shoot and root tissue biomasses were weighed fresh (FW), and their dry weight (DW) was recorded after being dried in an oven at 60°C. To determine root length, the washed roots were placed on transparent Perspex trays with a water film, and pictures were taken with a scanner (Epson, model Expression 1000 XL). The WinRHIZO software was used to calculate the length of the digitalized sample roots.

### Collection of root exudates

Just before the harvest of the maize plants from the rhizoboxes, moist sorption filters (5 mm Ø, filter paper: MN815, Macherey-Nagel, Düren, Germany) were placed on the surface of roots growing along the root observation window of the rhizoboxes, as outlined by Haase et al. (2008). Sampling was done for each rhizobox by applying the sorption filters in triplicate in subapical root zones (1–2 cm behind the root tip) and basal root zones (5–6 cm behind the root tip). To establish a negative control, sorption filters were placed on the soil where no visible root growth was present (soil without root contact). After a 4-hour incubation, the sorption filters from each of the rhizoboxes were collected and stored at -20°C (Neumann et al., 2014). Acetonitrile:H_2_O (1:1) was used to extract root exudates from sorption filters.

### Analysis of amino acids

A portion of 20 μl was extracted from a 0.6 ml sorption filter and combined with 15 μl of ACCQFLUOR REAG derivatization agent (Waters, Milford, MA, USA) along with 65 μl of borate buffer. This mixture was then incubated at 55°C for 10 minutes. Afterwards, 400 μl of acetonitrile:H_2_O (1:4) was added, based on modifications to the method described by Cohen and Michaud (1993). The identification of specific amino acids (glutamic acid, asparagine, serine, glutamine, glycine, threonine, histidine, alanine, proline, cysteine, tyrosine, methionine, isoleucine, leucine, and phenylalanine) was carried out using HPLC-MS on a Velos LTQSystem (Thermo Fisher Scientific, Waltham, MA, USA) equipped with an ACCUTAGTM column (Waters, Milford, MA, USA) with 4 μm particle size and dimensions of 150 mm × 3.9 mm. External standards were employed for comparison. A gradient elution method was employed utilizing (A) ammonium formate:methanol:H_2_O (40:9:60) and (B) acetonitrile (Windisch et al., 2021).

### DNA extraction, amplicon library preparation, and sequencing

Genomic DNA was extracted from the rhizosphere soil and root material by usingthe DNeasy PowerLyzer PowerSoil Kit (Qiagen). The universal primer pair 799f (Chelius and Triplett, 2001) and 1193r (Bodenhausen et al., 2013), which targets the V5-V7 region of the bacterial 16S rRNA gene, was used to characterize the bacterial community (Weisburg et al., 1991). The primer pair ITS1F/ITS2R was used to amplify the fungal internal transcribed spacer (ITS) rRNA region (White et al., 1990). The PCR protocols used were described in (Francioli et al., 2021). The obtained amplicons were sequenced on an Illumina MiSeq instrument with 2 × 300 base pair kits by LGC Genomics GmbH, Berlin, Germany. Demultiplexing was performed with Illumina bcl2fastq 2.17.1.14 software following the clipping of barcode and sequencing adapters. Only sequences with a quality score of above 20 were retained. DADA2 pipeline was used to determine amplicon sequence variants (ASV) from the raw sequences (Callahan et al., 2016). Alpha diversity metrics were calculated from the normalized sequence library, which was rarefied to 20,000 reads per sample for both microbial groups. Bacterial 16S ASV representative sequences were classified using the naive Bayesian classifier (Wang et al., 2007) for Silva 138. Representative sequences for fungal ITS ASVs were classified using the naive Bayesian classifier (Wang et al., 2007) against the Unite 9.0 reference database (Nilsson and Alexander, 2019). Amplicon sequence data were deposited at the European Nucleotide Archive under the BioProject accession number PRJEB73342 for bacterial and PRJEB73344 for fungal data. Furthermore, a *B. atrophaeus* ABi05 primer pair, designed from ABi05 genomic DNA sequences, was used for qPCR quantification of *B. atrophaeus* ABi05 DNA in the DNA samples according to the method described by Buttner et al. (2004). We only observed differences in the number of gene copies of *B. atrophaeus* ABi05 between the inoculated samples and the control for both rhizosphere and root compartments, while no significant differences were observed across the different treatments (**Supplementary Figure S2**).

### Data analysis

Univariate Analysis of Variance (ANOVA) followed by Tukey’s honestly significant difference (HSD) *post hoc* test was used to test for differences in soil and plant properties among the different treatments. All the variables were tested for normality using Shapiro-Wilk and Jarque-Bera tests and the equality of group variances was examined using Levene’s test. A log10 transformation was applied to all variables that did not meet the parametric assumptions. Correlations among the soil and plant traits were determined using Spearman’s rank correlation. Differences in bacterial and fungal ASV richness (ASV detected in each sample) between the different treatments were estimated using ANOVA followed by a Tukey’s HSD *post hoc* test. For β-Diversity analysis, we first calculated Bray-Curtis dissimilarities using the Hellinger transformation (square root transformation of relative abundances; Legendre and Gallagher (2001)). Permutational multivariate analysis of variances (PERMANOVA) based on the Bray-Curtis dissimilarity index was performed to analyze the effect of the plant-soil compartment, the nitrogen and phosphorus fertilization, and the *B. atrophaeus* inoculation on the bacterial and fungal community structure, using 999 permutations for each test. The soil and plant properties were fitted to the nonmetric multidimensional scaling ordination using the “envfit” function in the “vegan” package of R, and goodness-of-fit statistics (R^2^) were calculated with p-values based on 999 permutations. The linear discriminant analysis effect size (LEfSe) (Segata et al., 2011) was applied to identify biomarker taxa explaining differences between the bacterial and fungal microbiota due to the above mentioned experimental factors in both plant-soil compartments. All the data were analyzed with R version 3.6 (R Core Team, 2014).

## Results

### Plant biomass and root architecture

**Figure 1:**
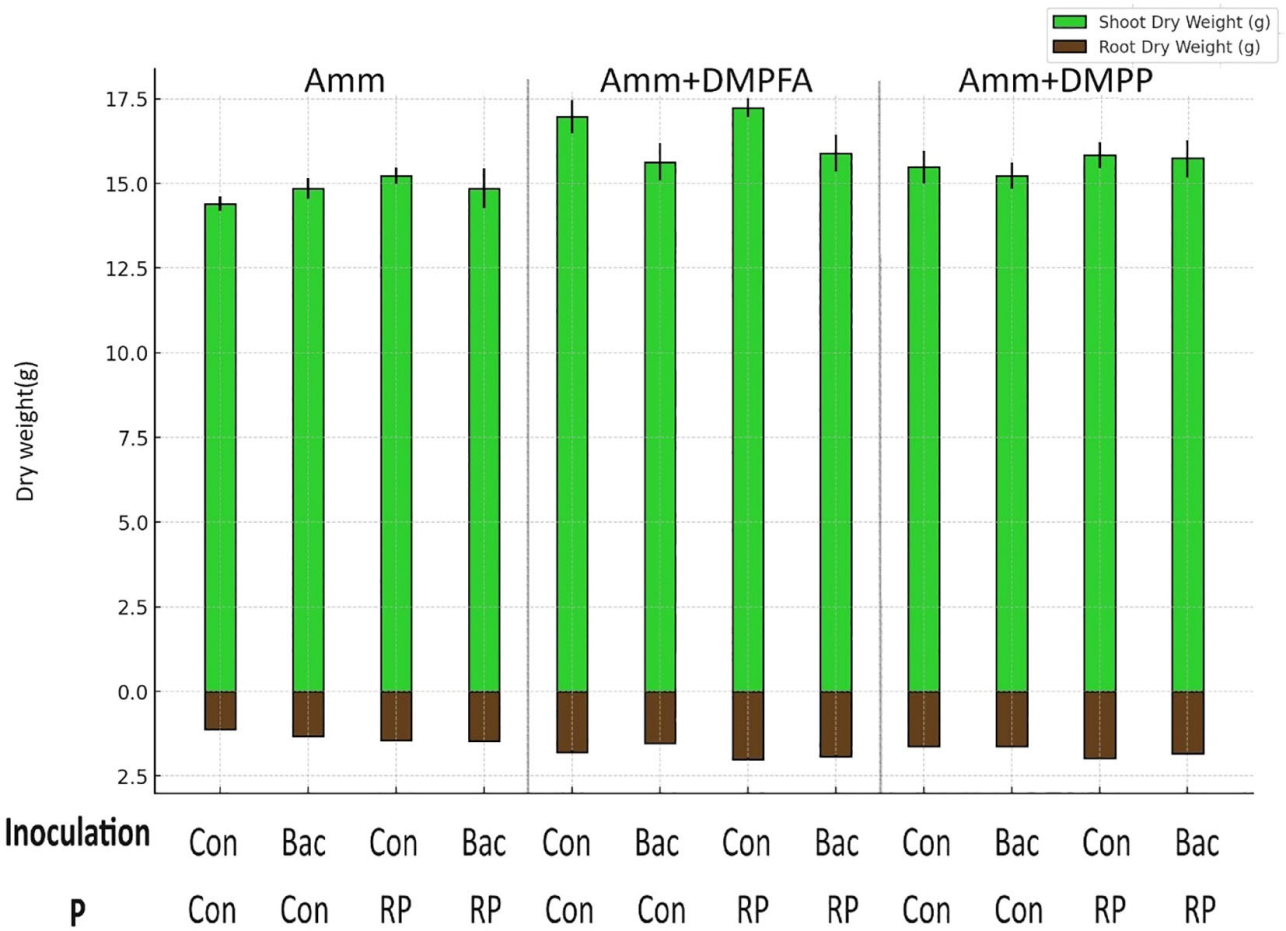
Root and shoot dry weight(g) in the treatments with ammonium, ammonium+ DMPFA and ammonium+ DMPP and phosphorus in two levels (Control and RP) and inoculation in two levels (Control and Bacillus atrophaeus) in maize (cv Ronaldinio) at 42 DAS. (Mean of four replications with standard error, Tukey test, p ≤ 0.05).

Shoot and root biomass were significantly affected by all three experimental factors, with nitrogen fertilization having the most pronounced effect (**Tables 1**, **2**). Notably, the use of DMPFA increased the total maize biomass by 13% compared to the control treatment (**Table 1**; **Figure 1**). RP fertilization and *B. atrophaeus* inoculation also increased plant biomass, as shown in **Table 2**. DMPFA and RP had a significant effect on root length<0.2 mm diameter, and they significantly increased this trait compared to the control, while no effect was found with *B. atrophaeus* inoculation. An increase in the root length of the roots with a diameter of 0.2 to 0.6 mm was observed with the employment of Nis, RP, and *B. atrophaeus*. A similar result was observed for the root with a diameter larger than 0.6 mm. DMPFA and *B. atrophaeus* significantly increased the total root length, but RP had no effect (**Supplementary Figure S3**).

**Table 1:** Morphological and physiological response in maize (cv Ronaldinio) at 42 DAS (day after sowing) due to employing nitrification inhibitors compared with ammonium.

		Nis	
	Amm	Amm+DMPFA	Amm+DMPP
Shoot fresh weight (g)	53.21 b	56.8 a	53.34 b
Shoot dry Biomass(g)	14.83 c	16.43 a	15.57 b
Root fresh weight (g)	15.88 b	20.28 a	20.82 a
Root dry weight(g)	1.34 c	1.81 a	1.76 b
Biomass (g)	16.17 c	18.24 a	17.33 b
Root diameter 0< L< 0.2mm	11391.6 b	14657.6 a	12175.9 b
Root diameter 0.2< L< 0.6mm	2995.8 ab	3357.2 ab	3745.6 a
Root diameter 0.6< L	816.6 b	787.7 b	883.6 a
Total root length (mm)	15204.1 b	18802.6 a	16805.2 b
Ca mg/g dm	3.34 b	3.62 a	3.69 a
Ca content mg per plant	49.56 b	59.43 a	57.36 a
Fe mg/g dm	0.078 b	0.085 a	0.081 ab
Fe content mg per plant	1.15 c	1.39 a	1.26 b
K mg/g dm	10.17 a	10.31 a	9.89 a
K content mg per plant	150.71 b	169.03 a	153.89 b
Mg mg/g dm	2.27 a	2.29 a	2.08 b
Mg content mg per plant	33.6 b	37.5 a	32.4 b
Mn mg/g dm	0.043 a	0.035 b	0.03 c
Mn content mg per plant	0.64 a	0.58 b	0.46 c
P mg/g dm	0.66 b	0.83 a	0.68 b
P content mg per plant	9.87 c	13.54 a	10.65 b
Zn mg/g dm	0.028 a	0.028 a	0.024 b
Zn content mg per plant	0.41 b	0.45 a	0.37 c
N (%)	1.25 b	1.32 a	1.33 a
NUE (%)	59.87 c	70.41 a	67.14 b
PUE (%)	3.57 b	4.53 a	3.59 b
Acid P.A. mg pNP/g Soil FM/hr	45.81 c	133.9 a	99.3 b
Alka P.A. mg pNP/g Soil FM/hr	64.62 b	89.07 a	94.28 a
Glutamic Acid (μg/cm Root)	0.13 c	0.20 b	0.24 a
Serin (μg/cm Root)	0.15 b	0.15 b	0.17 a
Asparagine (μg/cm Root)	0.17c	0.24 a	0.20 b
Glycine (μg/cm Root)	0.15 b	0.20 a	0.23 a
Glutamine (μg/cm Root)	0.34 b	0.53 a	0.54 a
Alanine (μg/cm Root)	0.10 b	0.11 b	0.14 a
Glucose (µg/cm root)	0.33 c	0.69 a	0.52 b
Sucrose (µg/cm root)	0.53 c	1.56 a	1.25 b
Rhizosphere pH	5.99 a	5.60 c	5.75 b

Within one trait, means followed by a common letter are not significantly different (Tukey test, p ≤ 0.05).

**Table 2:** Morphological and physiological response in maize (cv Ronaldinio) due to RP fertilization and *Bacillus atrophaeus* inoculation compared with control.

	Phosphorus		Inoculation	
	Con	RP	Con	Bac
Shoot fresh weight (g)	53.84 a	55.06 a	53.13 b	55.75 a
Shoot dry Biomass(g)	15.42 b	15.8 a	15.29 b	15.93 a
Root fresh weight (g)	18.58 a	19.41 a	18.91 a	19.08 a
Root dry weight(g)	1.50 b	1.77 a	1.58 b	1.7 a
Biomass(g)	16.93 b	17.57 a	16.87 b	17.62 a
Root diameter 0< L< 0.2mm	13536.6 a	11946.9 b	12227.1 a	13256.4 a
Root diameter 0.2< L< 0.6mm	3202.8 b	3529.66 a	2984.3 b	3748.2 a
Root diameter 0.6< L	791.6 b	867.1 a	806.2 b	852.5 a
Total root length(mm)	17531.0 a	16343.7 a	16017.6 b	17857.1 a
Ca mg/g dm	3.7 a	3.4 b	3.6 a	3.51 b
Ca content mg per plant	57.18 a	53.71 b	55.05 a	55.85 a
Fe mg/g dm	0.08 a	0.07 b	0.08 a	0.07 b
Fe content mg per plant	1.31 a	1.22 b	1.27 a	1.26 a
K mg/g dm	10.94 a	9.3 b	9.98 a	10.26 a
K content mg per plant	168.83 a	146.92 b	146.92 b	163.31 a
Mg mg/g dm	2.27 a	2.15 b	2.23 a	2.20 a
Mg content mg per plant	35.08 a	33.92 a	34.02 a	34.98 a
Mn mg/g dm	0.036 a	0.035 a	0.035 a	0.037 a
Mn content mg per plant	0.56 a	0.56 a	0.53 b	0.59 a
P mg/g dm	0.71 b	0.74 a	0.73 a	0.71 a
P content mg per plant	11.02 b	11.69 a	11.26 a	11.44 a
Zn mg/g dm	0.028 a	0.024 b	0.027 a	0.026 a
Zn content mg per plant	0.43 a	0.38 b	0.41 a	0.41 a
N (%)	1.32 a	1.28 a	1.31 a	1.29 a
NUE (%)	65.96 a	65.65 a	65.02 a	66.60 a
PUE (%)	–	–	3.77 b	4.02 a
Acid P.A. mg pNP/g Soil FM/hr	88.06 b	97.94 a	99.66 a	86.35 b
Alka P.A. mg pNP/g Soil FM/hr	81.47 a	83.83 a	81.57 a	83.74 a
Glutamic Acid (μg/cm Root)	0.19 a	0.19 a	0.14 b	0.24 a
Serin (μg/cm Root)	0.14 b	0.16 a	0.14 b	0.17 a
Asparagine (μg/cm Root)	0.22 a	0.18 b	0.16 b	0.23 a
Glycine (μg/cm Root)	0.18 b	0.21 a	0.19 a	0.19 a
Glutamine (μg/cm Root)	0.49 a	0.44 b	0.38 b	0.55 a
Alanine (μg/cm Root)	0.11 a	0.11 a	0.10 b	0.13 a
Glucose (µg/cm root)	0.30 b	0.72 a	0.52 a	0.5 a
Sucrose (µg/cm root)	0.69 b	1.53 a	1.16 a	1.06 a
Rhizosphere pH	5.84 a	5.71 b	5.78 a	5.77 a

Within one trait, means followed by a common letter are not significantly different (Tukey test, p ≤ 0.05).

### Root exudates, soil characteristics, and nutritional indicators

There were significant quantitative differences in the exuded compounds across the treatment groups. Notably, DMPFA and DMPP treatments resulted in a 195% and 135% increase in the exudation of sucrose, respectively, compared to the control plants (**Table 1**). RP also increases the exudate sucrose, while *B. atrophaeus* inoculation had no effect (**Table 2**; **Supplementary Figure S4**). Similarly, glucose concentration in the root exudates followed the same trend as sucrose. On the contrary, glutamine was shown to increase its concentration in the exudation profile along with Nis and *B. atrophaeus* application, while RP decreased its amount (**Supplementary Figure S5**). We lacked complete data on other root exudates due to their quantities being below detectable levels.

**Figure 2:**
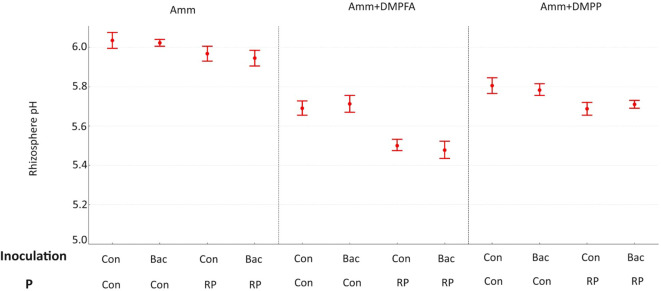
Rhizosphere pH 42 DAS for ammonium, ammonium+ DMPFA and ammonium+ DMPP with phosphorus in two levels (Con and RP) and inoculation in two levels (Con and Bacillus atrophaeus) in maize. (Tukey test, p ≤ 0.05).

The soil pH was also affected by the experimental treatments. Using Nis and RP caused a significant decrease in soil pH, while *B. atrophaeus* inoculation did not have any additional effect (**Figure 2**). Acid phosphatase activity was increased under Nis and RP treatment, while *B. atrophaeus* inoculation decreased it. Contrary, alkaline phosphatase was solely increased by Nis.

**Figure 3:**
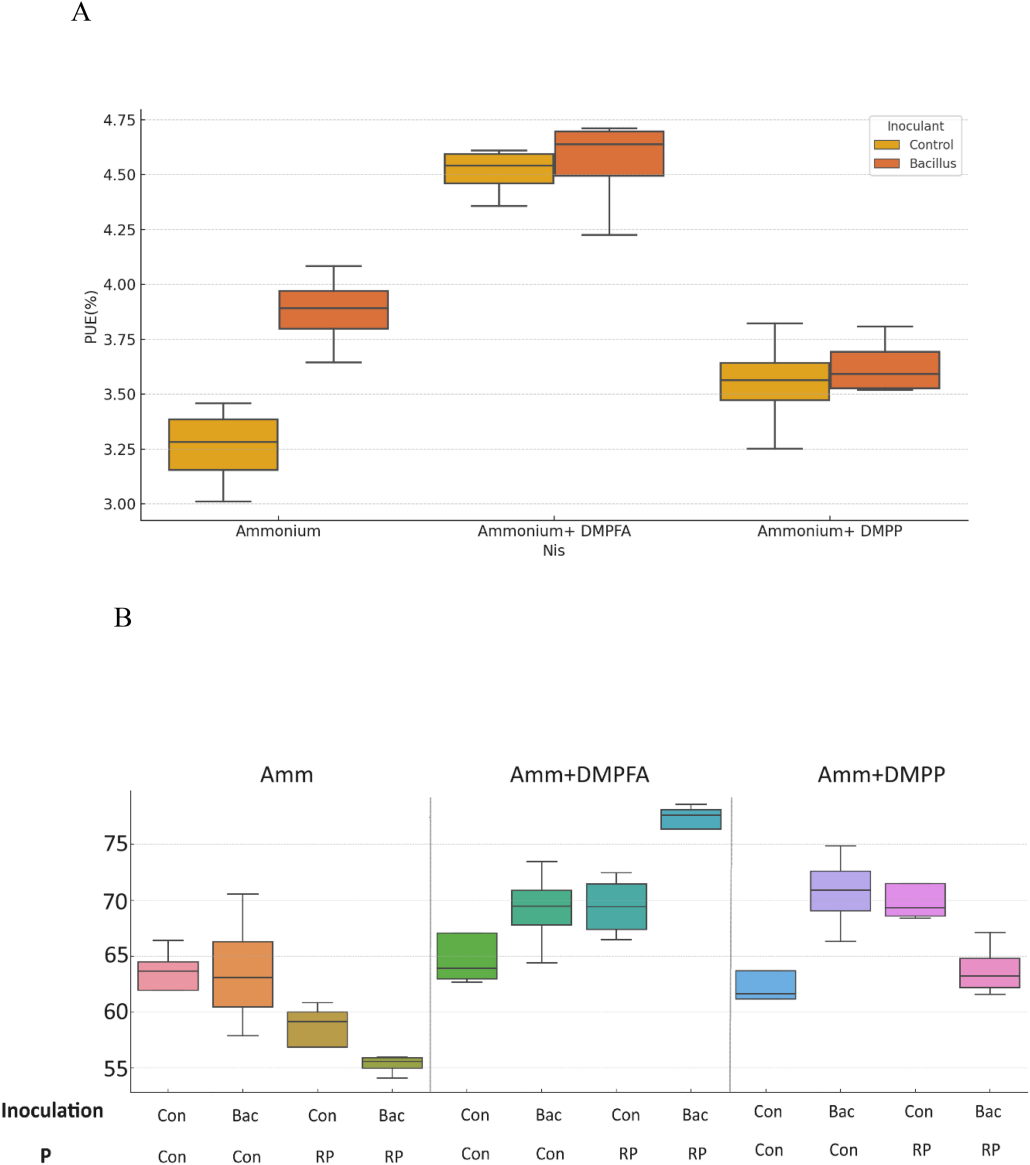
**(A)** Phosphorus use efficiency (PUE%) and **(B)** Nitrogen Use efficiency (NUE%) for ammonium, ammonium+ DMPFA and ammonium+ DMPP with inoculation in two levels (Con and Bacillus atrophaeus) in maize (cv Ronaldinio) at 42 DAS. (Tukey test, p ≤ 0.05).

P concentration showed a 26% increase in DMPFA compared with the control, but no effect on P concentration was observed for DMPP (**Table 1**). While the use of *B. atrophaeus* did not show significant differences, RP application also increased P concentration. Accordingly, P content mirrored the results related to P concentration. Control and Nis treatment exhibited no significant difference in K concentration. Although inoculation did not show a significant difference, RP had a significant impact on reducing K concentration. The concentration of Ca in Nis was significantly larger than control, while RP treatment reduced it. Conversely, the concentration of some cations decreased when Nis were employed. For instance, both Nis treatments led to a decrease in Mn concentration, with DMPFA and DMPP showing reductions of 19% and 30%, respectively, compared to the control. Notably, DMPP application led to a decrease in Mg and Zn concentrations by 8% and 14%, respectively. PUE was significantly greater than control in the DMPFA treatment, with a 27% increase (**Figure 3A**). RP and *B. atrophaeus* treatments did not affect NUE and PUE (**Table 2**). NUE was increased under Nis, with DMFA and DMPP showing an increase compared to control of 18% and 12%, respectively (**Figure 3B**).

### PCA and correlation analysis

**Figure 4:**
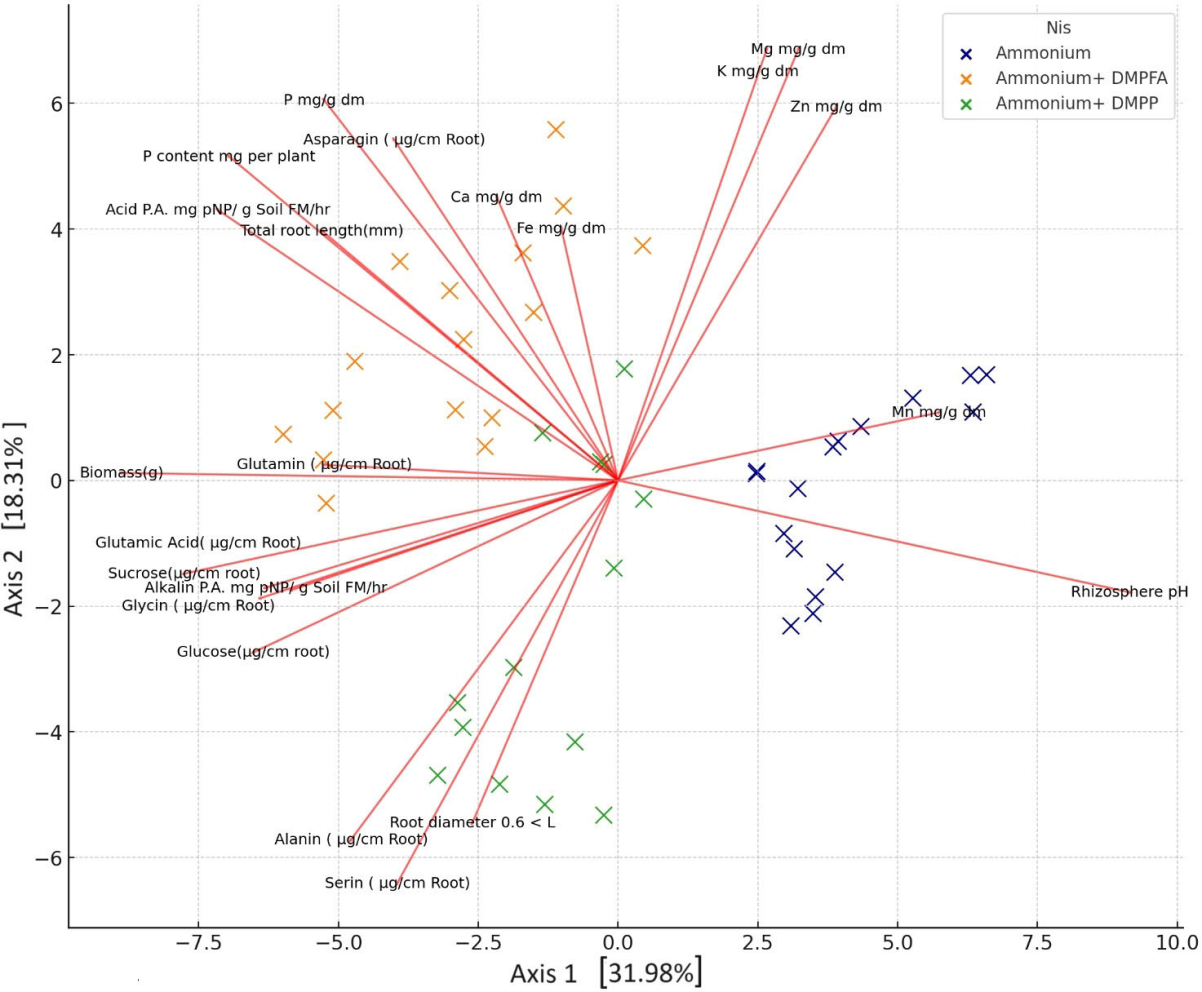
Principal component analysis (PCA) of the plant nutritional and rhizosphere traits based on the type of nitrification inhibitors.

**Figure 5:**
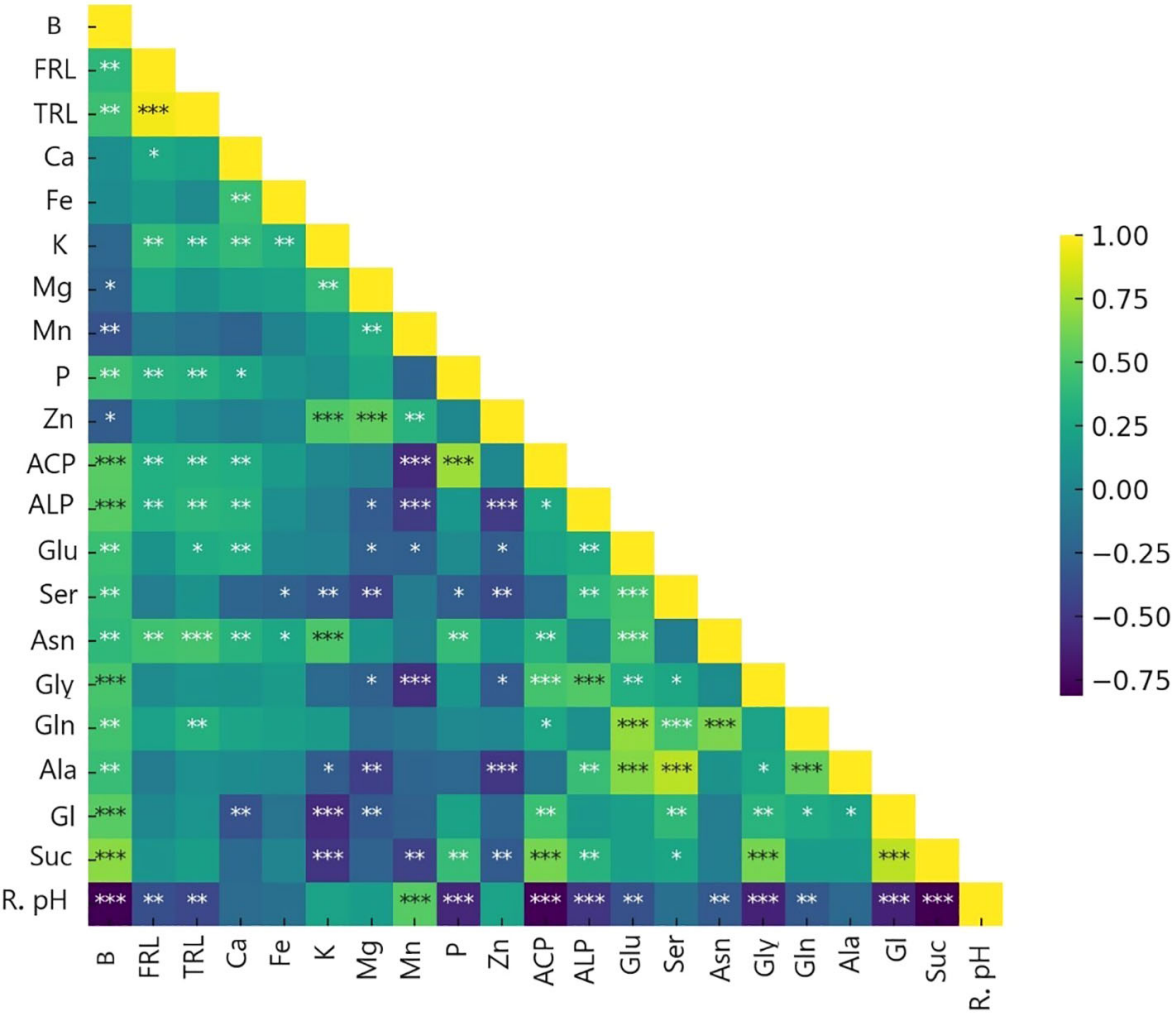
Heat map of Pearson’s correlation coefficients between plant traits (∗P< 0.05, ∗∗P< 0.01, ∗∗∗P< 0.001). B, biomass(g); FRL, fine root length (0<L<0.2mm); TRL, total root length (mm); Ca, Ca concentration mg/g dm; Fe, Fe concentration mg/g dm; K, K concentration mg/g dm; Mg, Mg concentration mg/g dm; Mn, Mn concentration mg/g dm; P, P concentration mg/g dm; Zn, Zn concentration mg/g dm; ACP, Acid phosphatase activity mg pNP/g Soil FM/hr; ALP, Alkaline phosphatase activity mg pNP/g Soil FM/hr; Glu, Glutamic acid µg/cm root; Ser, Serin µg/cm root; Asn, Asparagine µg/cm root; Gly, Glycine µg/cm root; Gln, Glutamine µg/cm root; Ala, Alanine µg/cm root; Gl, Glucose µg/cm root; Suc, Sucrose µg/cm root; R. pH, Rhizosphere pH.

By employing ammonium and Nis, separate clusters emerge, and traits are denoted by distinct lines that show in principal component analysis (PCA) of the plant nutritional and rhizosphere traits based on the type of nitrification inhibitors (**Figure 4**). **Figure 5** depicts how morphological, physical, nutritional status, and biochemical plant indicators are linearly related. Pearson’s correlation coefficients heatmap demonstrated a strong positive correlation between P content, Ca content, acid and alkaline phosphatase activity, glucose, and sucrose with biomass. The heatmap further indicates a strong negative correlation between rhizosphere pH and all indicators, except for the Zn content.

### Sequencing summary and microbial alpha-diversity

A median of 40,009 bacterial 16S rRNA gene reads and 22671 ITS fungal high-quality reads were recovered from the 72 rhizosphere samples (12 treatments × 2 compartments (root and rhizosphere) x 3 replicates), clustering in 3,025 bacterial and, 1,056 fungal ASVs. Overall, bacterial sequences were attributed to 30 bacterial phyla, 79 classes, 179 orders, 273 families, and 488 genera. Proteobacteria were the most prevalent phylum in all treatments comprising 36.9% of the total bacterial reads, followed by Actinobacteria (25.6% of reads) and Verrucomicrobiota (12.1%) (**Supplementary Figure S6**). Candidatus_Udaeobacter (7.5% of reads), Streptomyces (5.6% of reads), and Massilia (5.4% of reads) were the most abundant bacterial genera across all samples. Bacterial richness ranged from 320 to 694 ASVs, with rhizosphere samples showing larger values than root samples; no significant differences were observed between treatments (**Supplementary Figure S7**).

Fungal sequences were assigned to 10 phyla, 29 classes, 63 orders, 121 families, 233 genera. Ascomycota (85.6% of reads) was the dominant fungal phylum, followed by Basidiomycota (7.5% of reads) (**Supplementary Figure S6**). Fusarium (17.4%), Penicillium (5.5%), and Neocosmospora (5.3%) were the most representative fungal genera in our dataset. As expected, and similar to bacteria, fungal richness was higher in the rhizosphere than in the root (ranging from 23 to 248 ASVs), and no significant differences were observed between the investigated treatments (**Supplementary Figure S7**).

### Effect of the experimental variables on microbial β-Diversity

**Figure 6:**
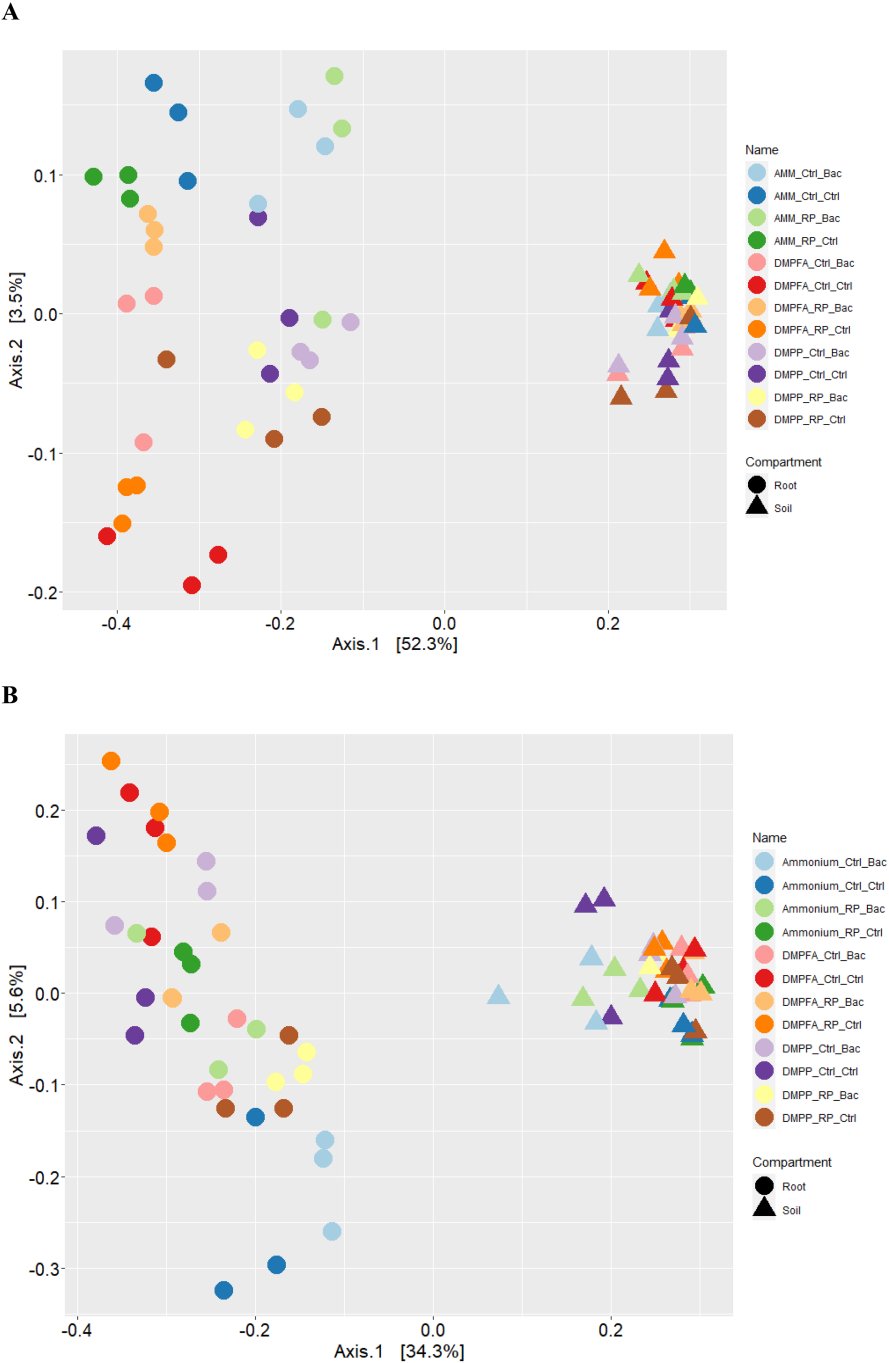
Principal coordinates analysis (PCoA) of the Bray-Curtis dissimilarity metrics of the **(A)** bacterial and **(B)** fungal communities detected in this study.

Compartmentalization emerged as the primary factor driving maize microbiota assembly, capturing 49.1% of the bacterial and 32.4% of fungal community variation (**Supplementary Table S1**). PERMANOVA analysis revealed that the rhizosphere and roots were characterized by distinct microbiota, and principal coordinate analysis (PCoA) clearly reflected this finding (**Figure 6**). Within each investigated compartment, we observed substantial and significant effects of different N and P fertilization, as well as *B. atrophaeus* inoculation, on the maize microbiota structure (**Tables 3**, **4**). The type of nitrogen fertilization represented the most critical factor for bacterial structure assembly in both compartments, accounting for 12.3% and 16.2% of the variation in the rhizosphere and root, respectively. The effect of differential N fertilization was also evident in the ordination plots, especially for the bacterial community associated with maize roots (**Figure 6**). *B. atrophaeus* inoculation significantly shaped the bacterial microbiome, capturing 6.6% of community variance in the root and 3.4% in the rhizosphere. Phosphorous fertilization marginally influenced the root bacterial community structure (3.2% of variation), while it had no effect on the rhizosphere bacteria assembly. We detected a significant interaction between N fertilization and *B. atrophaeus* inoculation, explaining approximately an additional 10% of the bacterial community variation in both compartments. Only in the root compartment we observe a significant interaction between N and P inoculation (6.6% of variation) and between *B. atrophaeus* inoculation and P fertilization (3.2%). A significant interaction among all three experimental variables on the bacterial community assembly was found in both compartments, accounting for 6.7% and 7.7% in the rhizosphere and root, respectively. These significant interactions suggest a differential response of the maize bacterial microbiota to the various experimental combinations established in the experiment.

**Table 3:** The effect of N fertilization, *Bacillus* inoculation and P fertilization on the bacterial community structure associated with the maize rhizosphere and root samples.

	Rhizosphere			Root		
Parameter	F	R^2^	P	F	R^2^	P
Nitrogen	2.621	0.123	0.001	4.115	0.162	0.001
*Bacillus*	1.465	0.034	0.030	3.333	0.066	0.001
Phosphorus	1.370	0.032	0.069	1.603	0.032	0.023
Nitrogen: *Bacillus*	2.159	0.101	0.001	2.388	0.094	0.001
Nitrogen: Phosphorus	1.096	0.051	0.219	1.682	0.066	0.005
*Bacillus:* Phosphorus	1.359	0.032	0.051	1.619	0.032	0.019
Nitrogen: *Bacillus*: Phosphorus	1.438	0.067	0.007	1.949	0.077	0.001

**Table 4:** The effect of N fertilization, *Bacillus* inoculation and P fertilization on the fungal community structure associated with the maize rhizosphere and root samples.

	Rhizosphere			Root		
Parameter	F	R^2^	P	F	R^2^	P
Nitrogen	2.454	0.106	0.001	2.502	0.105	0.001
*Bacillus*	1.842	0.040	0.003	1.848	0.039	0.003
Phosphorus	2.125	0.044	0.001	1.886	0.039	0.005
Nitrogen: *Bacillus*	2.464	0.106	0.001	1.927	0.081	0.001
Nitrogen: Phosphorus	1.553	0.067	0.003	2.910	0.122	0.001
*Bacillus:* Phosphorus	1.818	0.039	0.003	1.971	0.041	0.001
Nitrogen: *Bacillus*: Phosphorus	1.878	0.081	0.001	1.729	0.072	0.001

Similar to bacteria, the fungal community was primarily affected by N fertilization, capturing around 10.5% of community variation in both compartments. *B. atrophaeus* inoculation and P fertilization marginally affected fungal community structure, accounting for 4.4% variation in the rhizosphere and 3.9% variation in the root. An interaction between N fertilization and *B. atrophaeus* treatment was also found to be significant for fungal community assembly, explaining 10.6% and 8.1% of the variance in the rhizosphere and root, respectively. Fungal assembly was significantly influenced by the interaction between N and P fertilization, capturing 12.2% of variation in the roots and 6.7% in the rhizosphere. A significant interaction among all three experimental variables on fungal community structure was found 8.1% of community variance in the rhizosphere and 7.2% variation in the root.

Next, we assessed the contribution of measured edaphic properties to maize microbiota assembly. Soil pH, P, Fe, and Mn concentrations were significantly correlated with bacterial rhizosphere community assembly, while the bacterial root microbiome was significantly related to shifts in soil pH along with P, K, Mn, and Mg concentrations (**Supplementary Table S2**). Fungal community assembly in the rhizosphere was correlated with Zn concentration, whereas the maize root fungal population assembly dynamics were associated with Mg and Zn soil concentrations (**Supplementary Table S3**).

### Compositional phylogenetic shifts characterized microbial assembly in response to different fertilizers

**Figure 7:**
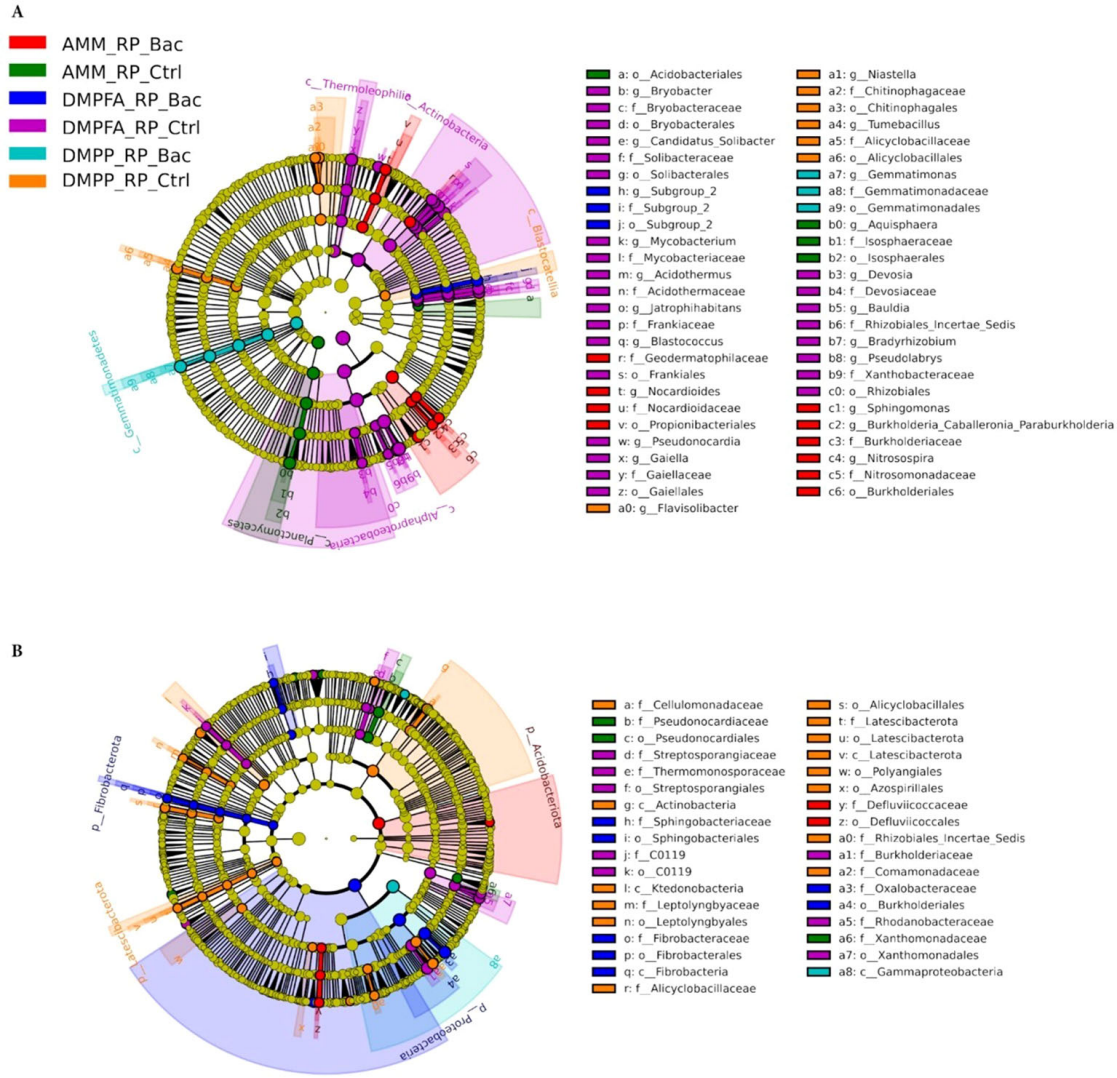
LEfSe analysis at multiple taxonomic levels comparing maize bacterial community structure between the treatment investigated in **(A)** rhizosphere and **(B)** root compartments. Cladogram illustrating the taxonomic groups, explaining the most variation among root communities. Each ring represents a taxonomic level, with phylum (p_), class (c_), order (o_) and family (f_) emanating from the center to the periphery. Each circle is a taxonomic unit found in the dataset, with circles or nodes shown in colors (other than yellow) indicating where a taxon was significantly more abundant.

Since all three experimental variables (N, P, and *B. atrophaeus* inoculation) significantly influenced maize microbial assembly, we focused our analysis on samples that received RP with all N and *B. atrophaeus* combinations. The evident differences observed in bacterial beta-diversity between the fertilization treatments were mainly reflected in substantial shifts in the composition of the bacterial communities. Importantly, biomarker analysis indicated that these compositional shifts in the bacterial microbiota occurring in both compartments (rhizosphere and root) due to the application of different fertilizers were highly phylogenetically clustered (**Figure 7**). Indeed, an enrichment of the Actinobacteria classes Actinomycetia and Thermoleophilia, and the Proteobacteria class Alphaproteobacteria, was observed in soil samples receiving DMPFA without *B. atrophaeus* inoculation. Conversely, samples related to DMPPA receiving the *B. atrophaeus* treatment showed a significant increase in bacterial taxa affiliated with the class Gemmatimonadetes (phylum Gemmatimonadota), while the treatment DMPP without *B. atrophaeus* showed an enrichment in bacterial members associated with the Acidobacteria class Blastocatellia. Interestingly, soil samples associated with the ammonium treatment exhibited a significant increase in the sole detected bacterial genera involved in the nitrification process, such as *Nitrosospira* and *Sphingomonas*, with their abundance significantly lower in the other treatments, especially in the DMPP treatment (**Supplementary Figure S8**). Regarding nitrification, we also identified taxa associated with the archaeal family *Nitrosotaleaceae*, but their proportion was marginal and did not differ across the treatments.

The bacteria community inhabiting maize roots also displayed strong phylogenetic shifts in the most dominant taxa due to the different treatments applied. For instance, root samples treated with ammonium and inoculated with *B. atrophaeus* showed an enrichment of bacteria associated with the Acidobacteriota phylum, while samples related to the treatment DMPFA + *B. atrophaeus* showed a significantly higher proportion of the phyla Proteobacteria and Fibrobacteriota compared to the other treatments. Contrarily, bacterial members affiliated with the Latescibacteriota phylum and the class Actinobacteria (Actinobacteriota phylum) were significantly more abundant in the DMPP without *B. atrophaeus* inoculation, while such N treatment together with *B. atrophaeus* showed a significantly higher proportion of the class Gammaproteobacteria (phylum Proteobacteria).

**Figure 8:**
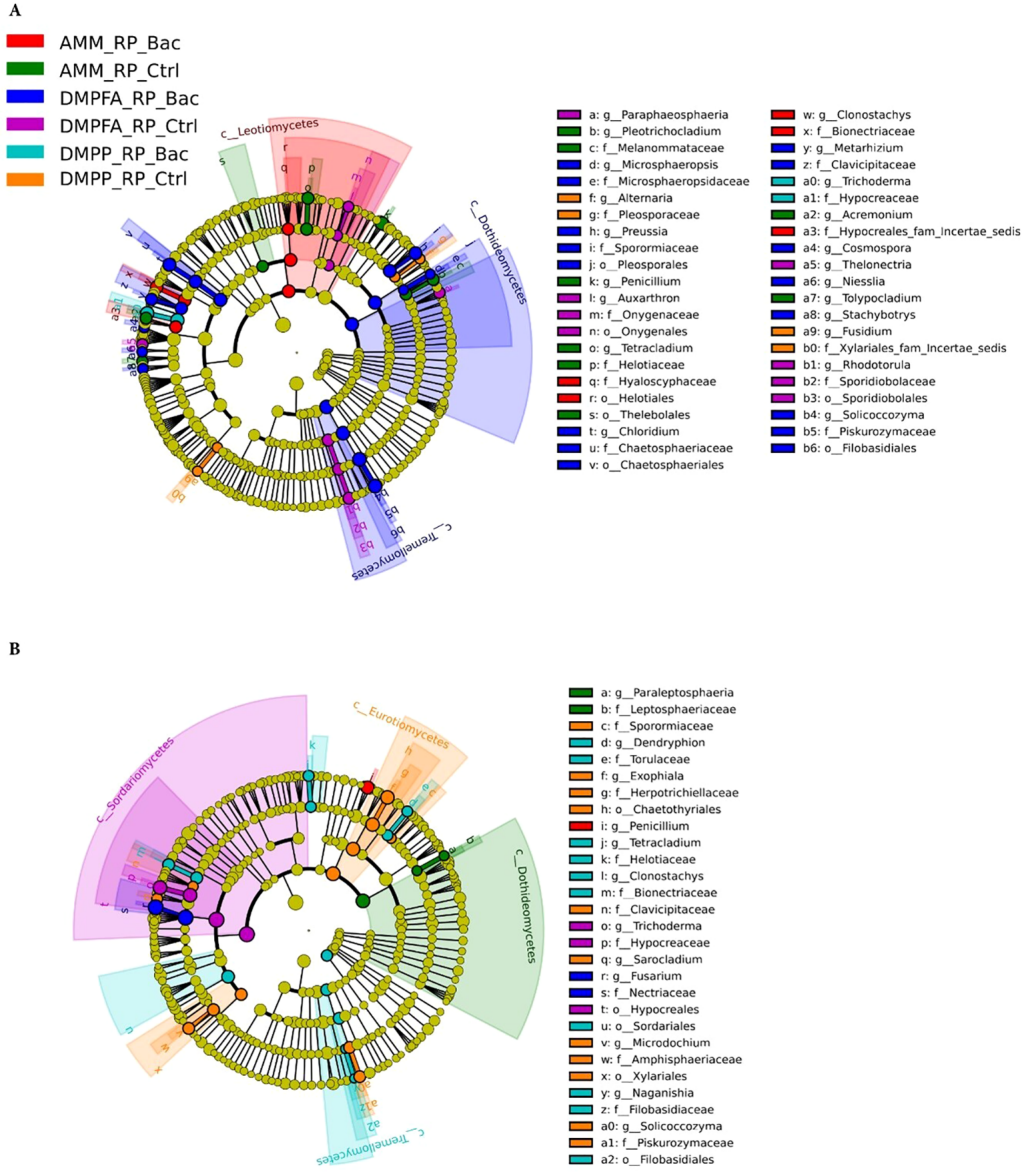
LEfSe analysis at multiple taxonomic levels comparing maize fungal community structure between the treatment investigated in **(A)** rhizosphere and **(B)** root compartments. Cladogram illustrating the taxonomic groups, explaining the most variation among root communities. Each ring represents a taxonomic level, with phylum (p_), class (c_), order (o_) and family (f_) emanating from the center to the periphery. Each circle is a taxonomic unit found in the dataset, with circles or nodes shown in colors (other than yellow) indicating where a taxon was significantly more abundant.

The fungal communities also exhibited a phylogenetic response to the different treatments, albeit not as pronounced as the bacterial population, reflecting the beta-diversity results (**Figure 8**). In the rhizosphere, for example, DMPFA + *B. atrophaeus* treatment was characterized by a significantly higher abundance of the classes Dothideomycetes (phylum Ascomycota) and Tremellomycetes (Basidiomycota). Taxa associated with the order Helotiales (Ascomycota) were enriched under ammonium treatment, especially with *B. atrophaeus* inoculation. Notably, fungi affiliated to the genus *Alternaria*, putative maize pathogens, were found significantly more abundant in the DMPP treatment, particularly without *B. atrophaeus* inoculation. Important shifts in the major fungal clades were also observed in the root compartment. Intriguingly, several Ascomycota classes were differentially enriched in the different treatments that did not involve *B. atrophaeus* inoculation. This was the case of Dothideomycetes in the ammonium treatment, Eurotiomycetes in DMPP and Sordariomycetes in DMPFA. Notably, taxa related to *Fusarium*, an important fungal genus encompassing important maize pathogenic fungi, were enriched under DMPFA. *Penicillium* and *Trichoderma*, two putative beneficial fungal genera, were found more abundant in the maize root under ammonium and DMPFA treatment, respectively.

## Discussion

The experiment showed how various Nis affect plant nutrient uptake, growth indicators, rhizosphere indicators, and soil microbial activity. While previous research (Mpanga et al., 2019a, b) has discussed the effects of microbial inoculation and Nis application, using DMPFA as a new nitrification inhibitor posed a challenge in this experiment.

Effects of Nis, *B. atrophaeus*, and RP on maize growth and nutrient uptake. The importance of roots less than 2 mm in diameter lies in their ability to take up water and nutrients and their rapid turnover rates (Block et al., 2006). Based on our results, the length of fine roots developed 29% more with DMPFA than with ammonium. Although the root length difference is not substantial for those roots with a bigger diameter, DMPFA shows a 24% increase in total root length compared to ammonium. The Nis likely resulted in a greater amount of root and shoot biomass due to the well-structured fine roots. In these scenarios, a larger root system that acidifies the soil might contribute to the solubilization of different phosphorus sources (such as Calcium-P, RP) and micronutrients. This would not only help plant species with limited capacity to mobilize nutrients through their roots, but also stimulate the development of mechanisms to solubilize sparingly soluble soil nutrients.

Ammonium fertilization further enhances the absorption of less soluble nutrients, such as P, Fe, Zn, and Mn, by inducing rhizosphere acidification. The study conducted by Ögüt et al. (2011) revealed that certain *B. atrophaeus* strains can boost proton release from roots due to ammonium, and also increase rhizosphere acid phosphatase activities in response to a lower rhizosphere pH. Rhizosphere acidification occurred in the treatment with a nitrification inhibitor in this experiment, despite the soil having a slightly acidic pH (6.4) prior to the experiment. Both Nis enhanced Ca, Fe, and P concentrations in the plant biomass, but there was no significant difference between K concentration between ammonium with and without Nis. Using RP decreased Ca and Fe concentration compared to the control, as was previously reported in another research (Martinengo et al., 2024; Mathan and Amberger, 1974). In a previous study, Dreyer et al. (2020) suggested that the use of Nis could cause decreased Mn availability in the bulk soil. In our experiment, we observed a 30% and 19% decrease in Mn concentration in DMPP and DPMFA compared to ammonium. Additionally, DMPP led to an 8% decrease in plant Mg concentration and a 14% decrease in Zn concentration compared to ammonium. In contrast, DMPFA did not exhibit any reduction in these cations. The negative correlation between ammonium and cation uptake is highlighted in some studies (Santos Silva et al., 2020; Bonomelli et al., 2021).

DMPFA application exhibited a significant difference in plant P concentration compared to ammonium alone, while no difference was observed between DMPP and ammonium. But P content in the plant was significantly larger for DMPFA and DMPP by 37% and 8% in comparison with ammonium, respectively. RP enhanced the plant P concentration in the treatment compared to the control. Previous research shows the increasing in P availability for maize with using nitrification inhibitors (Mpanga et al., 2019a). DMPFA induced a noteworthy 27% rise in PUE (**Figure 3**). However, there was no significant divergence between DMPP and ammonium alone. The NUE (%) in the DMPFA and DMPP treatments were significantly greater than that in the treatment with ammonium by 18% and 12% respectively, as expected. Utilizing DMP-based Nis has the potential to be a powerful strategy for reducing N losses (Guzman-Bustamante et al., 2022).

Phosphatases play a vital role in P acquisition for both plant roots and soil microbes. Phosphatase activity was significantly larger when Nis were used compared to ammonium alone. Compared with ammonium, an increasing 193% and 117% for acid phosphatase activity and 38% and 46% for alkaline phosphatase activity were observed when using DMPFA and DMPP, respectively. Increasing alkaline phosphatase activity and promote P release Our microbial analysis confirmed that the use of Nis resulted in greater activity in the root and microbial community, explaining the observed discrepancies. Application of Nis enhanced the secretion of amino acids from the roots. The increase in glutamine and asparagine in the DMPFA was remarkable.

### Effects of Nis, *B. atrophaeus*, and RP on maize microbiota

Soil microbial communities are profoundly influenced by various agricultural practices, including fertilization (Francioli et al., 2016; Guo et al., 2020; Lewin et al., 2024), microbial inoculation (Mawarda et al., 2020; Berg et al., 2021; Behr et al., 2023) and the application of crop protection products (Cesco et al., 2021; Li et al., 2023; Sim et al., 2023). The presence of fulvic acid also controls the type of microbial community in the soil (Piccolo et al., 2019). Our study elucidates the significant impact of three experimental variables—nitrogen inhibitors (Nis), *B. atrophaeus* inoculation, and RP addition—on the community composition of maize rhizosphere and root microbiota. Notably, Nis emerges as the primary determinant shaping bacterial and fungal communities across both soil-plant compartments. The documented influence of Nis application on soil microbiota assembly (Hou et al., 2023; Liu et al., 2024) underscores its pivotal role in mediating microbial dynamics. Given the notable alterations induced by Nis application in soil properties, including reduced pH and modified concentrations of soil nutrients and root-exuded metabolites in the rhizosphere, corresponding shifts in microbial composition were expected. Indeed, observed changes in microbiota assembly across treatments were primarily associated with variations in soil pH and alterations in the concentrations of P, K, Fe, Mg, and Mn due to Nis application. Soil pH is recognized as a key factor influencing the structure of rhizosphere and root microbiota across diverse soils and ecosystems (Bardelli et al., 2017; Tripathi et al., 2018; Shi et al., 2022). Similarly, soil nutrient concentrations and availability are recognized as significant drivers shaping rhizosphere and root microbiota, with their alterations holding substantial consequences for microbial assembly processes (Francioli et al., 2020; Custódio et al., 2022; Mang et al., 2023). Among microbial groups significantly responding to Nis application, a marked decrease in the relative abundance of ammonia-oxidizing bacteria (AOB) affiliated with the genus *Nitrosospira* was observed. *Nitrosospira* represents the primary nitrifying bacterial group detected in our study, capable of oxidizing ammonia, a crucial initial step in the nitrification process. Both Nis treatments employed in our experiment reduced Nitrosospira abundance compared to the control treatment (sole ammonium fertilization), with DMPP demonstrating greater efficacy in depleting bacterial taxa associated with this genus. These findings align with prior research indicating DMPP application significant reduction of AOB abundance (Lu et al., 2019; Bachtsevani et al., 2021) and provide novel insights into the DMPFA effect in diminishing the AOB soil community. The negative influence of Nis on the AOB community may occur through direct inhibition of specific nitrifying bacteria responsible for ammonia oxidation, altering microbial composition in the soil, or through indirect mechanisms involving modifications in nutrient availability, shifts in soil pH, and alterations in competitive microbial interactions (Hayatsu et al., 2008). In this line, it has been reported that the growth rate of AOB decreases with decreasing pH because NH_3_, the actual substrate of AOB, occurs only at very low concentrations when soil pH is less than 6, even at high ammonium concentrations (Suzuki et al., 1974). This observation further supports the negative indirect effect of Nis on the AOB community through decreased soil pH observed in our study.

The inoculation with *B. atrophaeus* and the addition of RP exhibited a significant, though modest, direct impact on the microbiota structure associated with the maize root and rhizosphere. Notably, *B*. *atrophaeus* successfully established and persisted in the maize rhizosphere (**Supplementary Figure S2**), consistent with other studies highlighting its strong capability to colonize the rhizosphere of maize and other crops affiliated with the *Poaceae* family (Andrić et al., 2020; Mahdi et al., 2021; Behr et al., 2023; Zuo et al., 2023). The establishment of B. *atrophaeus* in the rhizosphere of our maize plants significantly altered the indigenous soil microbial community composition and structure, aligning with recent findings (Behr et al., 2023; Hou et al., 2024), and this effect was more pronounced in the treatments where *B. atrophaeus* was coupled with Nis and RP use. Importantly, a significant interaction among all three experimental variables was observed, accounting for considerable variance in microbiota composition. This suggests a potential differential response of bacterial and fungal communities to the various treatment combinations investigated in this study. For instance, *B. atrophaeus* inoculation combined with DMPP and RP supplementation increased the abundance of Gemmatimonadetes in maize rhizosphere, an important bacterial class contributing to soil organic carbon cycling due to its metabolic strategies (Liu et al., 2020). Actinobacteria, notable for their role in promoting agricultural soil quality as remarkable organic matter decomposers (Strap, 2011), exhibited significantly higher abundance in soils treated with DMPFA and RP compared to other soils surveyed. The utilization of DMPFA, RP, and *B. atrophaeus* enriched the proportion of Proteobacteria in maize roots, the largest bacterial phylum known for its metabolic versatility and genetic diversity. Members of this phylum have generally been described as copiotrophic bacteria (Fierer et al., 2007; Lienhard et al., 2014; Francioli et al., 2016), which are fast-growing organisms that prefer nutrient-rich environments. Since the application of DMFPA and RP showed a significant increase in almost all the measured macro- and micronutrients, especially with the DMFPA application, it is likely that this treatment promoted the growth of Proteobacteria populations. Moreover, certain treatment combinations selectively promoted or diminished specific groups of soil microorganisms, which could be categorized as either beneficial or detrimental. For instance, taxa associated with putative beneficial fungal genera such as *Penicillium* and *Trichoderma* exhibited enrichment under the influence of ammonium and DMPFA treatments, respectively, when supplemented with RP. On the contrary, taxa related to *Fusarium*, an important fungal genus encompassing important maize pathogenic fungi, were enriched in the DMPFA and RP treatment. Our findings demonstrate that specific management practices can selectively enrich, promote, or suppress specific populations of soil microorganisms. This highlights the importance of furthering our understanding of how various management strategies impact soil and plant-associated microbial communities and the associated soil microbial processes. Such knowledge is crucial for developing sustainable agricultural production systems that optimize crop yields.

Despite being used at low application rates, Nis may be affected in the rhizosphere by the synergistic effects of fulvic acid as a chelator and bio stimulant, along with DMP as a chelator and nitrification inhibitor. Further experiments are needed to explore this.

## Conclusion

DMPFA has the potential to increase PUE, P content, and P concentration in maize, whereas DMPP only increases P content. Nis, especially DMPFA, may influence the soil microorganisms responsible for releasing Mg, Zn, Fe. As Nis alters microbial activity, it can affect the availability of cation negatively. While Nis enhanced plant biomass and growth indicators, there were some decreases in cation concentrations that should be considered in stress and diseased conditions. The interaction between Nis and inoculation, as well as P in its sparingly soluble form, affects the biotic and abiotic indices in maize. Further experimentation with DMPFA across different soil conditions and plant species is necessary to fully understand its potential.

## Data Availability

The datasets presented in this study can be found in online repositories. The names of the repository/repositories and accession number(s) can be found in the article/**Supplementary Material**.
