# Supplementary material for: Optimizing nitrogen fertilization in maize: the impact of nitrification inhibitors, phosphorus application, and microbial interactions on enhancing nutrient efficiency and crop performance
Source: Front Plant Sci. 2024 Oct 2;15:1451573. doi: 10.3389/fpls.2024.1451573 (PMC11479917; doi:10.3389/fpls.2024.1451573)
Supplement: Supplementary file 1 [file DataSheet1.docx]

Supplementary Figures


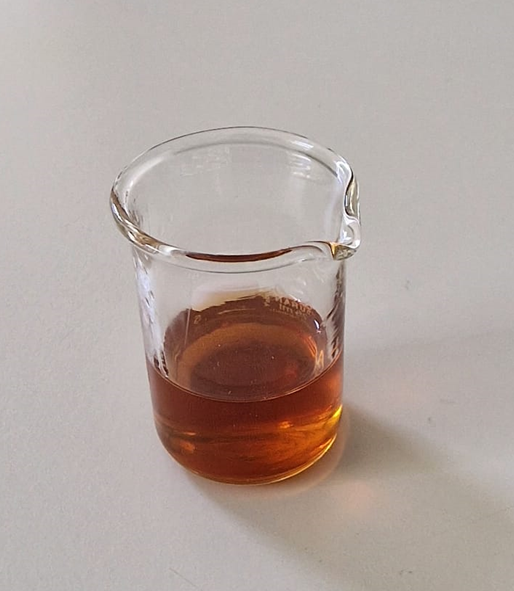


Figure S1. DMPFA (Dimethyl pyrazole fulvic acid) produced in the University of Hohenheim (Photo: Ali Malakshai Kurdestani)

**A**


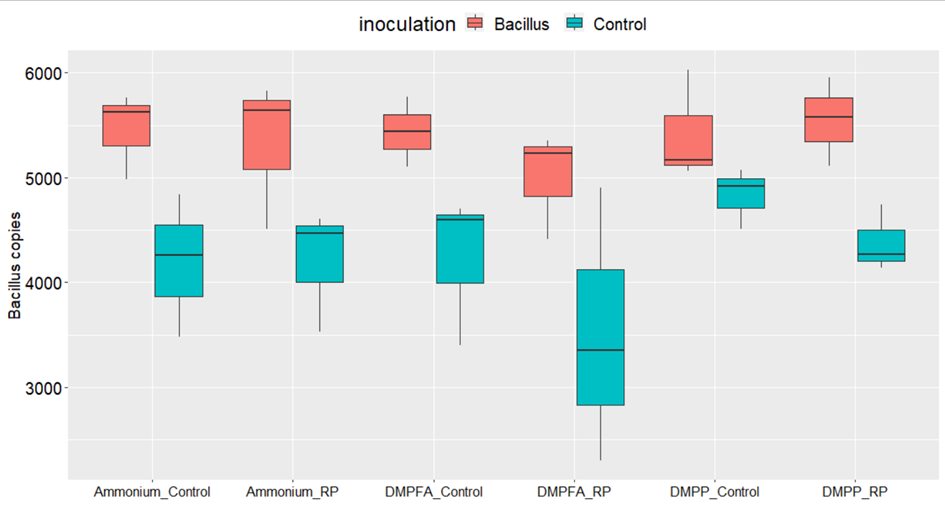


**B**


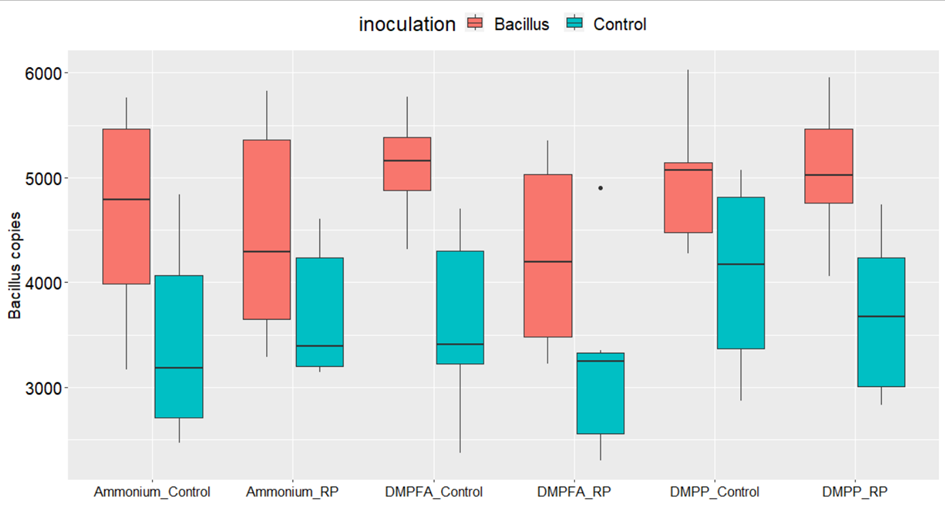


Figure S2. Boxplots showing the absolute abundances of Bacillus atrophaeus in (a) soil and (b) root samples across the different treatments obtained by qPCR.

**
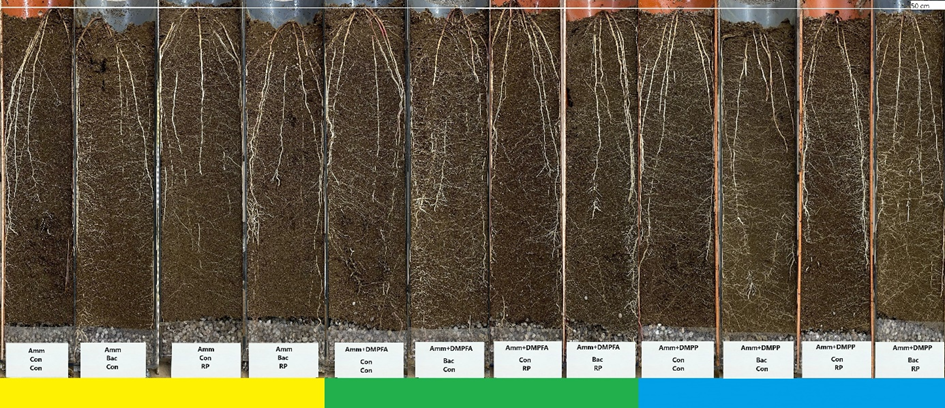
**

Figure S3. The root architecture varies for the treatments: ammonium (yellow), Amm+ DMPFA (green), and Amm+ DMPP (blue). This picture demonstrates how the root structure differs between Nis and ammonium. (Photo: Ali Malakshahi Kurdestani)


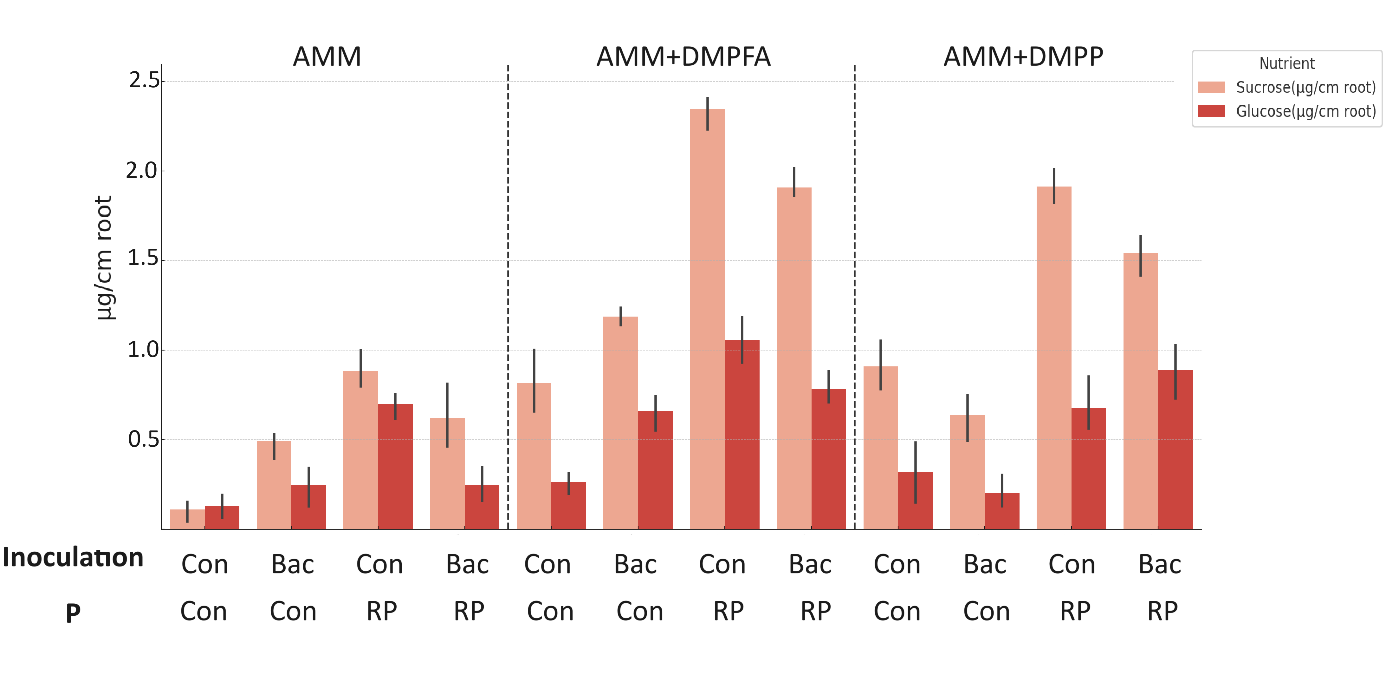


Figure S4. Sugars µg/ cm roots for ammonium, ammonium+ DMPFA and amm+ DMPP with phosphorus at two levels (Con and Rock-P) and inoculation at two levels (Con and Bacillus atrophaeus) in maize (cv Ronaldinio) at 42 DAS. Means of four replications, Tukey t test with error bar.


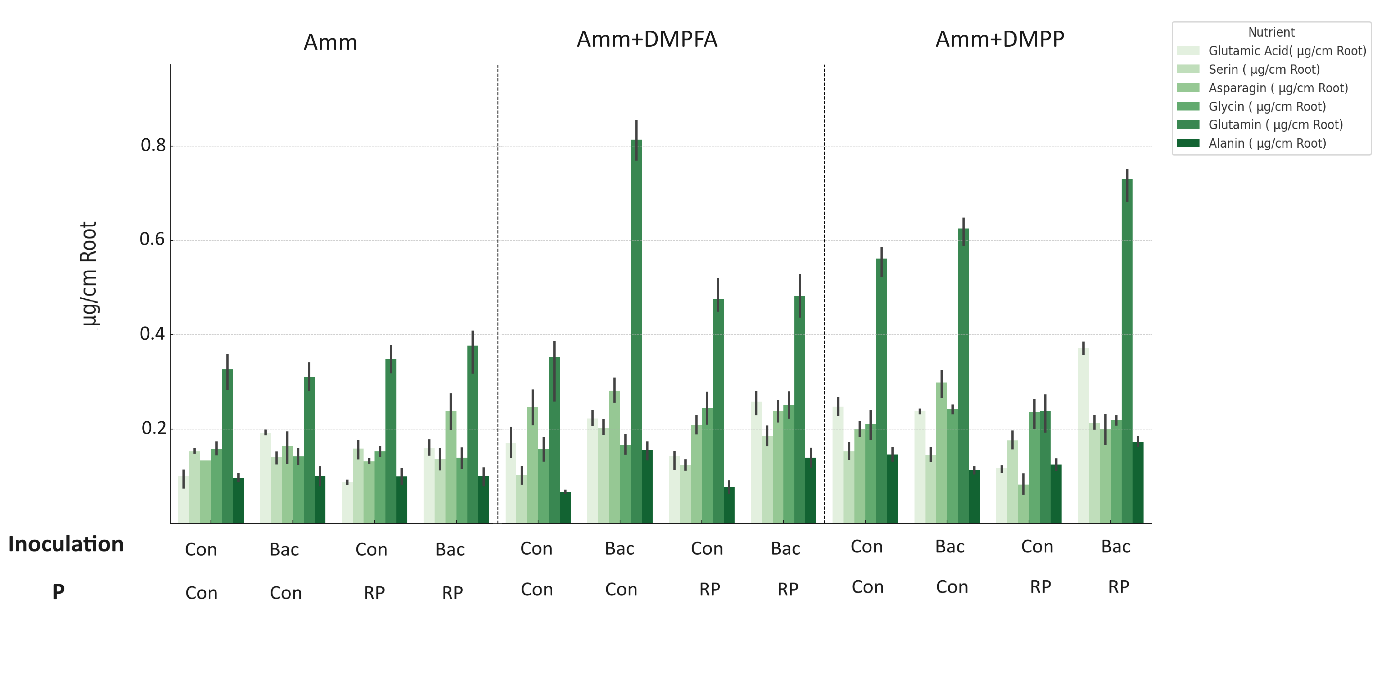


Figure S5. Amino acids µg/ cm roots for ammonium, ammonium+ DMPFA and amm+ DMPP with phosphorus in two levels (Con and Rock-P) and inoculation in two levels (Con and Bacillus atrophaeus) in maize (cv Ronaldinio) at 42 DAS. Means of four replications, Tukey honest test with error bar.

**A**


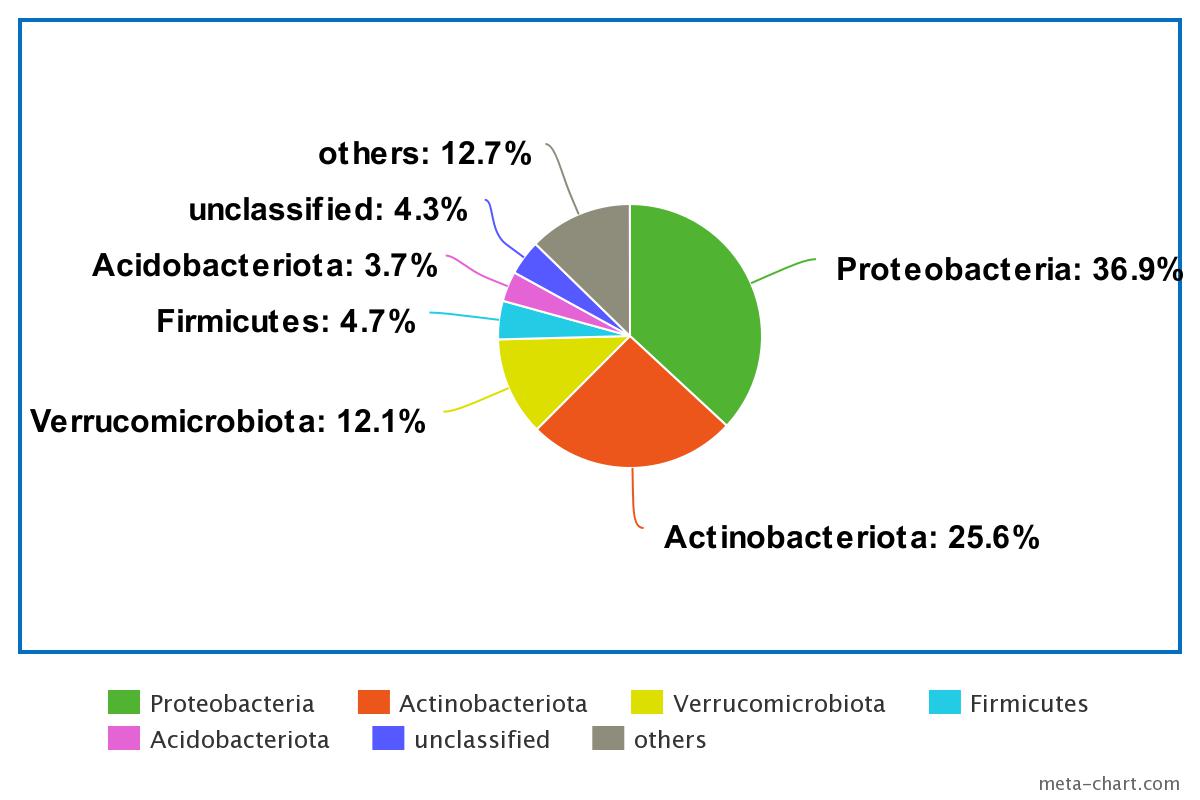


**B**
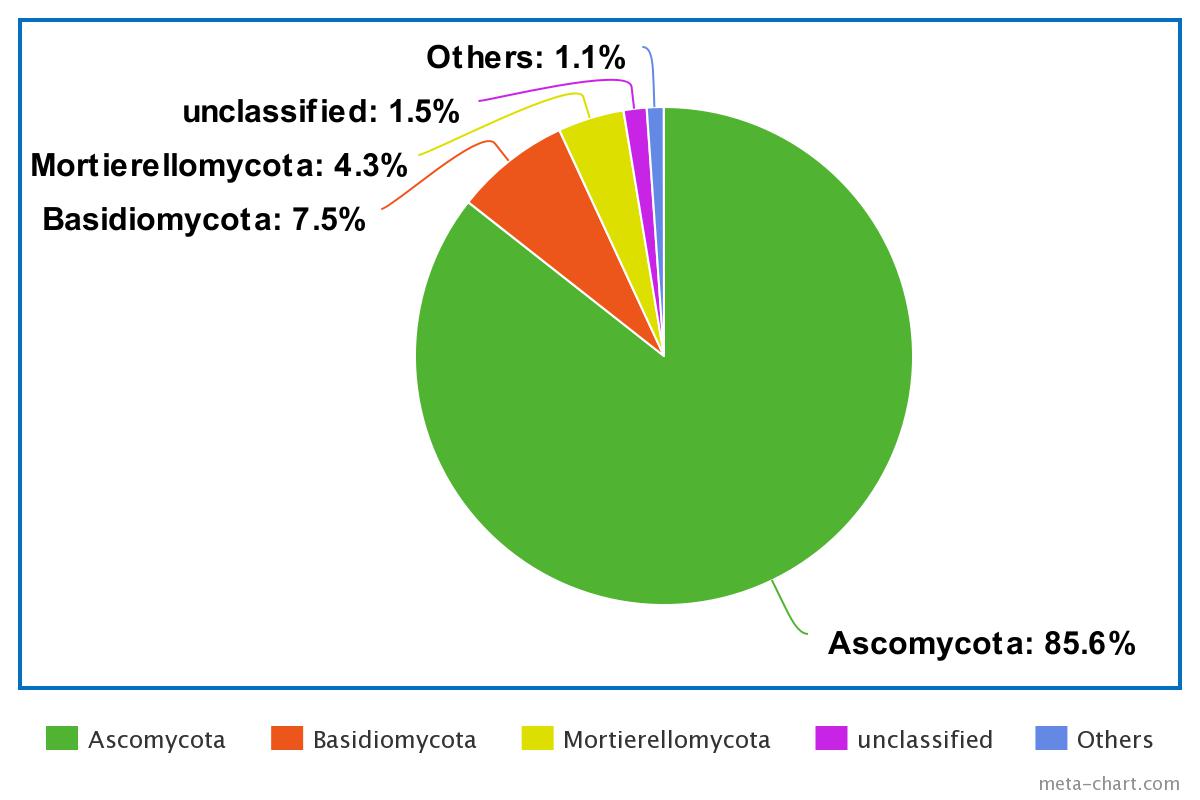


Figure S6. Relative abundances of the major phyla detected within the (A) bacterial and (B) fungal datasets.

**A**


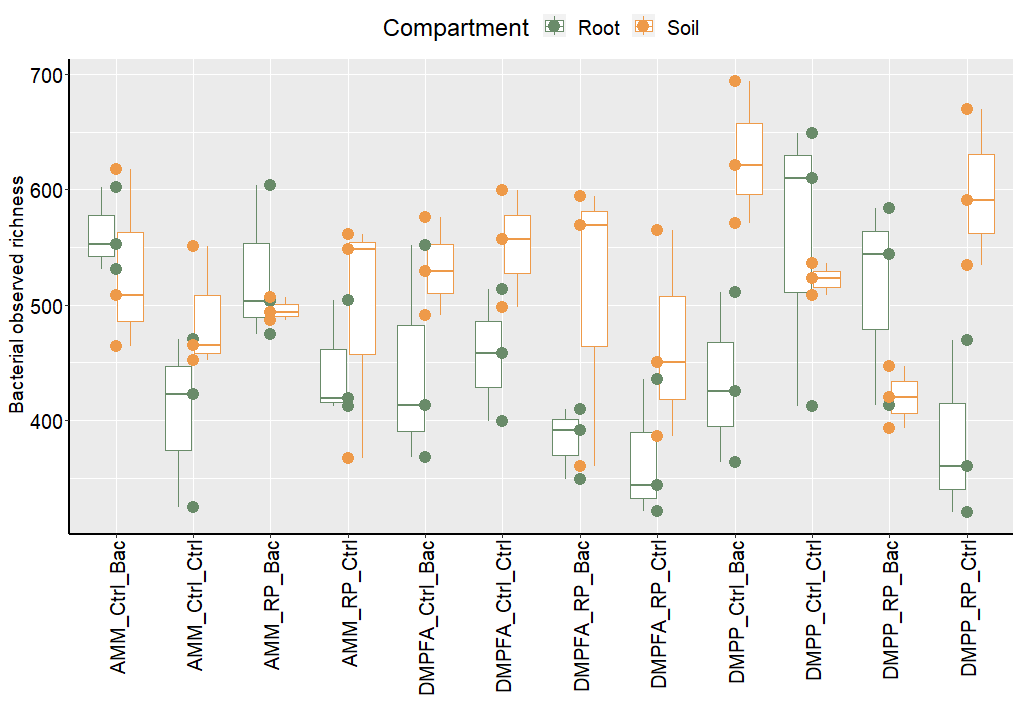


**B**
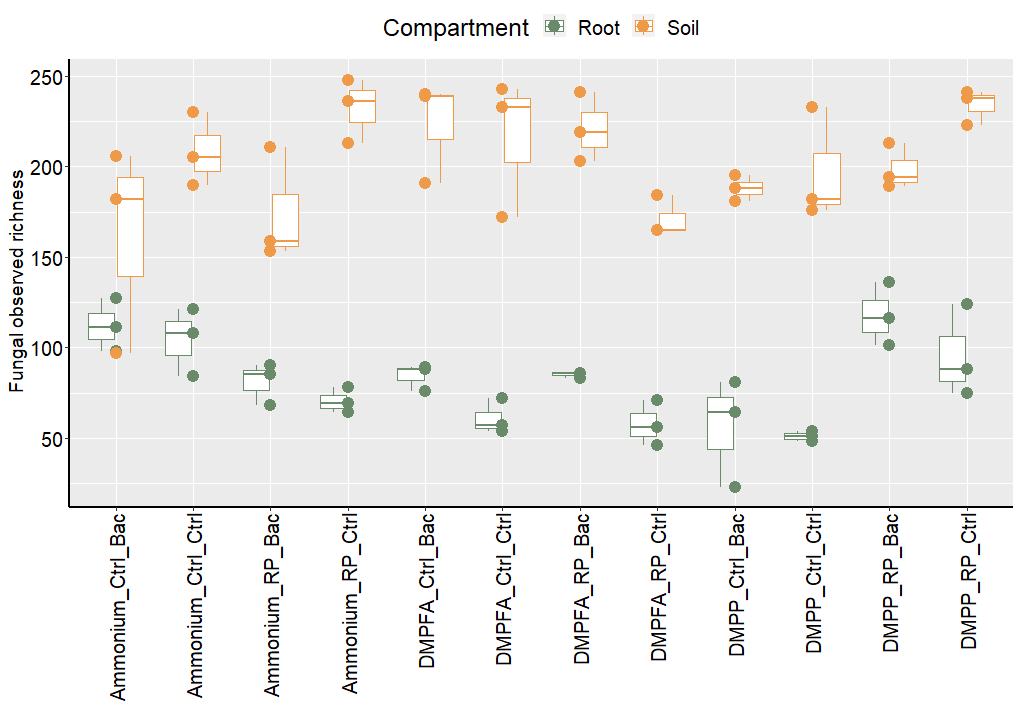


Figure S7. Box plots of the observed richness of (A) bacterial and (B) fungal communities in the rhizosphere and root compartments across the experimental treatments studied.


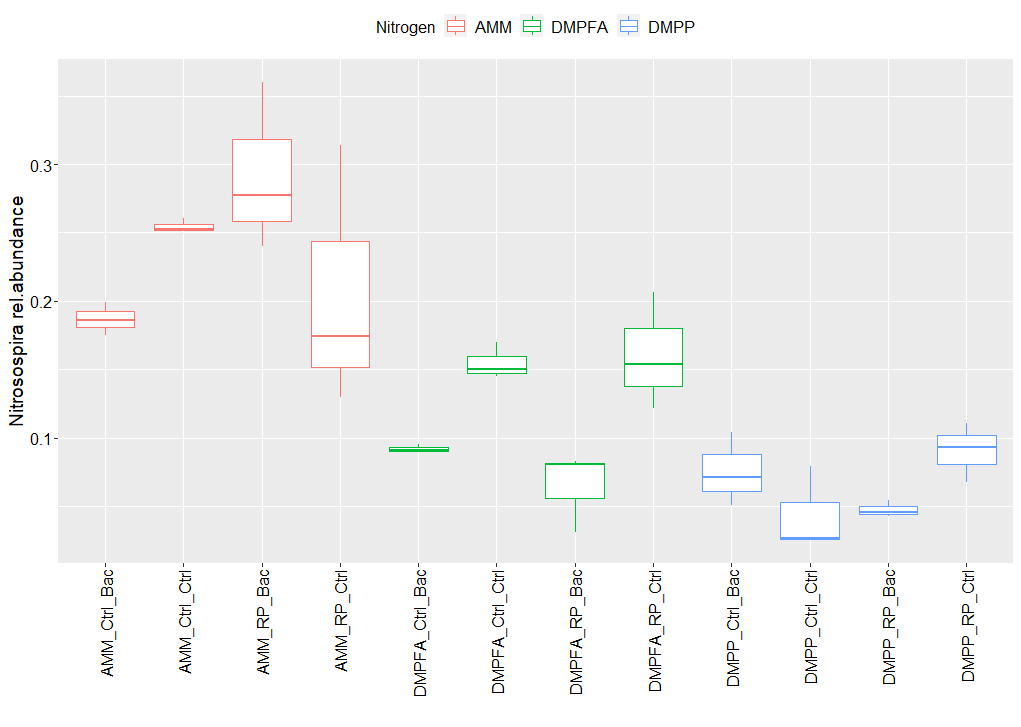


Figure S8. Relative abundance of the bacteria genus Nitrosospira in the soil samples.

Supplementary tables

**Table S1.** The effect of plant compartment, N fertilization, *Bacillus* inoculation and P fertilization on the bacterial and fungal community structure in our samples.

|  | Bacteria | | | | Fungi | | |  |
| --- | --- | --- | --- | --- | --- | --- | --- | --- |
| Parameter | F | R^2^ | P | F | | R^2^ | P | |
| Compartment | 10.392 | 0.491 | 0.001 | 2.45 | | 0.324 | 0.001 | |
| Nitrogen | 3.264 | 0.283 | 0.003 | 3.961 | | 0.041 | 0.003 | |
| Bacillus inoculation | 2.13 | 0.133 | 0.01 | 1.967 | | 0.0139 | 0.01 | |
| Phosphorous | 1.650 | 0.008 | 0.117 | 2.193 | | 0.015 | 0.039 | |

**Table S2.** Goodness-of-fit statistics (R^2^) of environmental variables fitted to the nonmetric multidimensional scaling ordination of bacterial community structure in maize rhizosphere and root samples. Only significant variables are reported.

|  | Rhizosphere | | |  | | Root | | | |
| --- | --- | --- | --- | --- | --- | --- | --- | --- | --- |
| Parameter | R^2^ | P |  | |  | | R^2^ | P |  |
| Soil pH | 0.19 | 0.032 |  | |  | | 0.193 | 0.025 |  |
| P cont | 0.180 | 0.044 |  | |  | | 0.206 | 0.029 |  |
| Mn | 0.178 | 0.042 |  | |  | | 0.174 | 0.039 |  |
| Mg | 0.1251 | 0.107 |  | |  | | 0.185 | 0.037 |  |
| Fe | 0.164 | 0.040 |  | |  | | 0.1388 | 0.08 |  |

**Table S3.** Goodness-of-fit statistics (R^2^) of environmental variables fitted to the nonmetric multidimensional scaling ordination of fungal community structure in maize rhizosphere and root samples. Only significant variables are reported.

|  | Rhizosphere | | |  | | Root | | | |
| --- | --- | --- | --- | --- | --- | --- | --- | --- | --- |
| Parameter | R^2^ | P |  | |  | | R^2^ | P |  |
| Zn | 0.160 | 0.041 |  | |  | | 0.158 | 0.048 |  |
| Mg | 0.0246 | 0.679 |  | |  | | 0.153 | 0.047 |  |
